# Supplementary material for: Microwave Dielectric Response of Bovine Milk as Pregnancy Detection Tool in Dairy Cows
Source: Sensors (Basel). 2024 Apr 25;24(9):2742. doi: 10.3390/s24092742 (PMC11086119; doi:10.3390/s24092742)
Supplement: Supplementary file 1 [file sensors-24-02742-s001.zip › sensors-2865698-supplementary.pdf]

SUPPORTING INFORMATION 1: **Physiological and Microwave Dielectric fitting parameters measured weekly for individual tracked cows among three different reproductive states: Non-Pregnant, Insemination, and Pregnancy.**

Table S1A: Physiological parameters (Somatic cell count -SCC, Fat %, protein%, lactose% and Milk Fat Globule Average diameter) measured weekly for n=12 different cows at different reproductive states (Non-pregnant-NP, during inseminations and during confirmed pregnancy). The not measured days correspond mostly to Saturdays, an official holiday in Israel.

n = 1

week	DATE	cow number	DIM (days in milk)	STATE	SCC (10^3/mL)	Fat %	protein %	lactose %	MFG Average diameter
1	13.5.20	4027	55	NON-PREGNANT	44	3.05	3.52	4.71	3.3
2	20.5.20	4027	62	NON-PREGNANT	44	2.67	3.19	4.78	3.7
3	27.5.20	4027	69	NON-PREGNANT	31	2.8	3.36	4.8	3.57
4	3.6.20	4027	76	NON-PREGNANT	31	2.57	3.08	4.82	3.7
5	10.6.20	4027	83	NON-PREGNANT	31	2.78	3.17	4.85	3.37
6	17.6.20	4027	90	NON-PREGNANT	15	3.1	3.49	4.83	3.15
7	24.6.20	4027	97	INSEMINATION 1	22	3.26	3.42	4.94	3.15
8	1.7.20	4027	104	INSEMINATION 1	22	3.68	3.48	4.96	3.56
9				INSEMINATION 1					
10	15.7.20	4027	118	INSEMINATION 2	18	3.74	3.29	5.02	3.51
11	21.7.20	4027	124	INSEMINATION 2	18	3.43	3.5	5	3.51
12	27.7.20	4027	130	INSEMINATION 2	18	3.49	3.27	5.05	4.18
13	05.08.20	4027	139	INSEMINATION 3	18	3.72	3.64	4.99	3.6
14	12.08.20	4027	146	INSEMINATION 3	19	3.65	3.44	5	NO
15	19.08.20	4027	153	INSEMINATION 3	19	3.7	3.49	5	3.39

16	26.08.20	4027	160	INSEMINATION 4	19	3.69	3.34	4.93	3.53
17	02.09.20	4027	167	INSEMINATION 4	19	3.68	3.35	4.95	2.92
18	09.09.20	4027	174	INSEMINATION 4	19	3.98	3.34	5	3.38
19	16.09.20	4027	181	INSEMINATION 5	58	3.64	3.26	4.93	3.3
20				INSEMINATION 5					
21	1.10.20	4027	196	INSEMINATION 6	58	3.99	3.3	4.99	3.8
22	7.10.20	4027	202	INSEMINATION 6	58	3.76	3.42	4.92	3.5
23	14.10.20	4027	209	INSEMINATION 6	58	4.06	3.32	5.04	4.07
24	22.10.20	4027	217	INSEMINATION 6	38	4.08	3.28	5.03	3.6
25	28.10.20	4027	223	INSEMINATION 6	38	3.87	3.24	4.98	3.3
26	04.11.20	4027	230	INSEMINATION 6	38	4.08	3.28	4.92	3.4
27	11.11.2020	4027	237	INSEMINATION 7	47	4.04	3.23	5.07	3.49
28	26.11.2020	4027	252	INSEMINATION 7	47	4.14	3.57	4.94	3.16
29	3.12.2020	4027	259	INSEMINATION 7	47	3.5	3.71	4.62	3.3
30	09.12.2020	4027	265	INSEMINATION 7	47	3.94	3.26	5.09	4.03
31	16.12.2020	4027	272	INSEMINATION 7	25	3.56	3.56	5.01	3.47
32	23.12.2020	4027	279	INSEMINATION 8	25	3.99	3.26	5.09	3.9
33	30.12.2020	4027	286	INSEMINATION 8	25	4.08	3.36	4.99	3.2
34	06.1.2021	4027	293	INSEMINATION 8	25	4.58	3.37	5.04	3.68
35	13.1.2021	4027	300	INSEMINATION 8	48	3.6	3.43	4.94	3.3
36	27.1.2021	4027	314	INSEMINATION 8	48	4.29	3.33	5.14	3.87
37	04.02.2021	4027	322	INSEMINATION 8	48	4.28	3.34	5.11	3.43
38	11.02.2021	4027	329	INSEMINATION 8	48	4.11	3.35	5.09	3.6
39	17.02.2021	4027	335	INSEMINATION 8	30	3.91	3.36	5.12	3.7
40	23.02.2021	4027	341	INSEMINATION 9	30	4.77	3.51	5.1	4
41	04.03.2021	4027	350	INSEMINATION 9	30	4.58	3.39	5.13	3.9
42	11.03.2021	4027		INSEMINATION 9					
43	18.03.2021	4027	364	INSEMINATION 9	47	3.91	3.25	4.82	3.7

44	25.03.2021	4027	371	INSEMINATION 10	47	4.39	3.59	5.07	3.75
45	05.04.2021	4027	382	INSEMINATION 10	47	4.67	3.61	4.6	4.7
46	12.04.2021	4027	389	INSEMINATION 10	37	5.03	3.41	5.17	2.8
47	19.04.2021	4027	396	INSEMINATION 10	37	4.15	3.65	4.88	3.37
48	26.04.2021	4027	403	INSEMINATION 11	37	4.06	3.55	5.09	4
49	05.05.2021	4027	412	INSEMINATION 11	37	3.81	3.49	4.86	3.28
50	19.05.2021	4027		INSEMINATION 11					
51	26.05.2021	4027	426	INSEMINATION 12	49	3.93	3.71	4.87	3.41

n=2

week	DATE	cow number	DIM (days in milk)	state	SCC (10^3/mL)	% Fat	Protein %	Lactose %	MFG Average diameter
1	13.5.20	4032	43	NON-PREGNANT	35	3.05	3.29	5.03	3.65
2	20.5.20	4032	50	NON-PREGNANT	35	2.25	3.09	5.05	4.2
3	27.5.20	4032	57	NON-PREGNANT	60	3.23	2.89	5.12	3.94
4	3.6.20	4032	64	NON-PREGNANT	60	2.8	3.05	5.03	4.5
5	10.6.20	4032	71	NON-PREGNANT	60	2.79	3.06	5.11	4.07
6	17.6.20	4032	78	NON-PREGNANT	49	2.64	2.92	5.09	4
7	24.6.20	4032	85	INSEMINATION 1	49	3.07	3.1	5.15	4.8
8	1.7.20	4032	92	INSEMINATION 1	49	3.49	3.14	5.21	3.94
9				INSEMINATION 1					
10	15.7.20	4032	106	INSEMINATION 1	37	3.74	3.04	5.22	4.23
11	21.7.20	4032	112	INSEMINATION 1	37	3.47	3.37	5.13	4.49
12	27.7.20	4032	118	INSEMINATION 1	37	3.31	3.27	5.17	4.76
13	05.08.20	4032	127	INSEMINATION 1	37	3.46	3.57	5.1	3.95
14	12.08.20	4032	134	INSEMINATION 1	53	3.27	3.28	5.15	4.49

15	19.08.20	4032	141	INSEMINATION 1	53	2.92	3.14	5.02	4.1
16	26.08.20	4032	148	INSEMINATION 2	53	3.66	3.13	5.19	3.8
17	02.09.20	4032	155	INSEMINATION 2	53	3.59	3.31	5.14	3.52
18	09.09.20	4032	162	INSEMINATION 2	53	3.62	3.19	5.1	4.8
19	16.09.20	4032	169	INSEMINATION 2	124	3.65	3.01	5.2	3.75
20				INSEMINATION 2					
21	1.10.20	4032	184	INSEMINATION 2	124	3.51	3.43	5.01	3.6
22	7.10.20	4032	190	INSEMINATION 2	124	3.85	3.18	5.13	4.1
23	14.10.20	4032	197	INSEMINATION 2	124	3.76	3.1	5.16	3.68
24	22.10.20	4032	205	INSEMINATION 2	57	3.54	3.49	4.92	4.2
25	28.10.20	4032	211	INSEMINATION 2	57	3.37	3.16	5.06	2.74
26	04.11.20	4032	218	INSEMINATION 3	57	3.5	3.59	4.96	4.19
27	11.11.2020	4032	225	INSEMINATION 3	87	3.83	3.22	5.14	4.33
28	26.11.2020	4032	240	INSEMINATION 3	87	3.41	3.57	5.01	3.61
29	3.12.2020	4032	247	INSEMINATION 3	87	2.85	3.53	4.94	4.11
30	09.12.2020	4032	253	INSEMINATION 4	87	3.49	3.24	5.1	4.4
31	16.12.2020	4032	260	INSEMINATION 4	92	4.94	3.2	5.11	3.99
32	23.12.2020	4032	267	INSEMINATION 4	92	3.94	3.23	5.14	4.4
33	30.12.2021	4032	274	INSEMINATION 5	92	3.71	3.28	5.07	3.65
34	06.1.2021	4032	281	INSEMINATION 5	92	3.78	3.14	5.13	3.51
35	13.1.2021	4032	288	INSEMINATION 5	51	3.9	3.49	5.06	3.8
36	27.1.2021	4032	302	INSEMINATION 5	51	3.73	3.38	5.07	3.41
37	04.02.2021	4032	310	INSEMINATION 6	51	3.52	3.29	5.01	3.47
38	11.02.2021	4032	317	INSEMINATION 6	51	3.75	3.24	5.11	3.6
39	17.02.2021	4032	323	INSEMINATION 6	49	3.55	3.2	5.02	3.8
40	23.02.2021	4032	329	INSEMINATION 6	49	3.74	3.32	5.01	3.9
41	04.03.2021	4032	338	INSEMINATION 6	49	3.96	3.45	5.01	4.19

42	11.03.2021	4032		INSEMINATION 6					
43	18.03.2021	4032	352	INSEMINATION 6	82	3.88	3.25	5.18	3.9
44	25.03.2021	4032	359	INSEMINATION 6	82	3.65	3.47	4.95	3.95
45	05.04.2021	4032	370	INSEMINATION 6	82	3.67	3.61	4.48	3.49
46	12.04.2021	4032	377	INSEMINATION 6	45	3.46	3.32	4.57	
47	19.04.2021	4032	384	INSEMINATION 7	45	3.54	3.43	5.04	4.4
48	26.04.2021	4032	391	INSEMINATION 7	45	3.25	3.43	4.9	3.97
49	05.05.2021	4032	400	INSEMINATION 7	45	4.04	3.37	5.08	3.2
50	19.05.2021	4032		INSEMINATION 7					
51	26.05.2021	4032	414	INSEMINATION 7	66	3.65	3.29	4.84	4.37

n = 3

week	DATE	cow number	DIM (days in milk)	state	SCC (10^3/mL)	% Fat	Protein %	Lactose %	MFG Average diameter
1	26.11.2020	3802	39	NON-PREGNANT	12	4.09	3.79	4.89	4.35
2	03.12.2020	3802	46	NON-PREGNANT	12	3.95	3.72	4.5	5
3	09.12.2020	3802	52	NON-PREGNANT	12	2.82	3.81	4.36	4.56
4	16.12.2020	3802	59	NON-PREGNANT	13	4.07	3.9	4.63	4.38
5	23.12.2020	3802	66	NON-PREGNANT	13	3.79	3.55	4.82	4.19
6	30.12.2020	3802	73	INSEMINATION 1	13	3.81	3.47	5.04	4.1
7	06.1.2021	3802	80	INSEMINATION 1	13	3.69	3.78	4.76	3.87
8	13.1.2021	3802	87	INSEMINATION 1	8	2.47	3.81	4.31	3.43
9	27.1.2021	3802	101	INSEMINATION 2	8	3.75	3.5	4.81	4.64
10	04.02.2021	3802	109	INSEMINATION 2	8	3.82	3.49	5.01	4.4
11	11.02.2021	3802	116	INSEMINATION 2	8	4.37	3.44	4.79	3.41

12	17.02.2021	3802	122	INSEMINATION 2	12	4.36	3.43	4.82	3.5
13	23.02.2021	3802	128	INSEMINATION 2	12	3.99	3.87	4.62	3.6
14	04.03.2021	3802	137	INSEMINATION 3	12	4.12	3.72	4.7	4.35
15	11.03.2021	3802		INSEMINATION 3					
16	18.03.2021	3802	151	INSEMINATION 3	23	3.85	3.67	4.68	3.86
17	25.03.2021	3802	158	INSEMINATION 3	23	4.08	3.94	4.73	3.42
18	05.04.2021	3802	169	INSEMINATION 4	23	3.5	4.02	4.27	3
19	12.04.2021	3802	176	INSEMINATION 4	20	4.64	3.46	4.88	3.69
20	19.04.2021	3802	183	INSEMINATION 5	20	4.79	3.57	4.35	3.2
21	26.04.2021	3802	190	INSEMINATION 5	20	3.58	3.51	4.95	4
22	05.05.2021	3802	199	INSEMINATION 5	20	4.24	3.46	4.85	3.43
23	19.05.2021	3802		INSEMINATION 6					
24	26.05.2021	3802	213	INSEMINATION 6	36	4.42	3.56	4.76	4.51

n =4

week	DATE	cow number	DIM (days in milk)	state	SCC (10^3/mL)	% Fat	Protein %	Lactose %	MFG Average diameter
1	23.12.2020	3971	33	NON-PREGNANT	32	3.45	3.41	5.01	4.21
2	06.1.2021	3971	47	NON-PREGNANT	32	3.42	3.37	4.98	4.59
3	13.1.2021	3971	54	NON-PREGNANT	7	2.72	3.28	5.04	4.08
4	27.1.2021	3971	68	NON-PREGNANT	7	3.61	3.29	5.03	4.54
5	04.02.2021	3971	76	NON-PREGNANT	7	3.38	3.37	4.91	4.3
6	11.02.2021	3971	83	NON-PREGNANT	7	3.53	3.38	4.93	3.4
7	17.02.2021	3971	89	NON-PREGNANT	7	3.64	3.44	4.92	4.2
8	23.02.2021	3971	95	NON-PREGNANT	7	3.67	3.5	4.76	3.12
9	04.03.2021	3971	104	INSEMINATION 1	7	3.45	3.26	4.99	4.21

10	11.03.2021	3971		INSEMINATION 1					
11	18.03.2021	3971	118	INSEMINATION 1	29	3.74	3.48	4.88	4.39
12	25.03.2021	3971	125	INSEMINATION 1	29	3.64	3.84	4.81	3.71
13	05.04.2021	3971	136	INSEMINATION 2	29	3.68	3.69	4.59	4.2
14	12.04.2021	3971	143	INSEMINATION 2	17	3.63	3.38	4.69	3.8
15	19.04.2021	3971	150	INSEMINATION 2	17	3.34	3.33	4.75	3.6
16	26.04.2021	3971	157	INSEMINATION 2	17	3.31	3.55	4.92	3.89
17	05.05.2021	3971	166	INSEMINATION 2	17	3.48	3.29	4.97	3.82
18	19.05.2021	3971		INSEMINATION 2					
19	26.05.2021	3971	180	INSEMINATION 3	19	3.56	3.48	4.95	4.21

n = 5

Week	DATE	cow number	DIM (days in milk)	state	SCC (10^3/mL)	% Fat	Protein %	Lactose %	MFG Average diameter
1	04.02.2021	3915	60	NON-PREGNANT	16	3.48	3.03	4.92	3.6
2	11.02.2021	3915	67	NON-PREGNANT	16	3.58	3	4.93	3.22
3	17.02.2021	3915	73	NON-PREGNANT	22	2.92	3	4.85	2.7
4	23.02.2021	3915	79	NON-PREGNANT	22	3.61	3.2	4.8	3.7
5	04.03.2021	3915	88	INSEMINATION 1	22	3.38	3.1	4.52	4.16
6	11.03.2021	3915		INSEMINATION 1					
7	18.03.2021	3915	102	INSEMINATION 1	46	3.56	3.04	4.8	3.8
8	25.03.2021	3915	109	INSEMINATION 1	46	3.4	3.4	4.82	3.73
9	05.04.2021	3915	120	INSEMINATION 1	46	2.94	3.41	4.42	2.6
10	12.04.2021	3915	127	INSEMINATION 1	20	3.76	3.12	4.94	3.24

11	19.04.2021	3915	134	INSEMINATION 1	20	3.62	3.05	4.91	3.3
12	26.04.2021	3915	141	INSEMINATION 1	20	4.04	3.47	4.84	3.69
13	05.05.2021	3915	150	INSEMINATION 2	20	3.47	3.12	4.96	3.75
14	19.05.2021	3915		INSEMINATION 2					
15	26.05.2021	3915	164	INSEMINATION 2	40	3.87	3.18	4.98	3.76

n = 6

week	DATE	cow number	DIM (days in milk)	state	SCC (10^3/mL)	% Fat	Protein %	Lactose %	MFG Average diameter
1	18.03.2021	3991	60	NON-PREGNANT	566	3.43	3.21	4.91	4.34
2	25.03.2021	3991	67	NON-PREGNANT	566	1.15	3.34	4.8	3.98
3	05.04.2021	3991	67	NON-PREGNANT	566	1.15	3.34	4.8	3.98
4	12.04.2021	3991	78	NON-PREGNANT	566	3.48	3.58	4.3	4.1
5	19.04.2021	3991	85	NON-PREGNANT	18	3.23	3.02	5	4.3
6	26.04.2021	3991	99	INSEMINATION 1	18	3.58	3.67	4.85	3.616
7	05.05.2021	3991	108	INSEMINATION 1	18	3.59	3.42	4.88	3.48
8	19.05.2021	3991		INSEMINATION 1					
9	26.05.2021	3991	122	INSEMINATION 2	13	3.14	3.27	4.73	4.6

n = 7

week	DATE	cow number	DIM (days in milk)	state	SCC (10^3/mL)	% Fat	Protein %	Lactose %	MFG Average diameter
1	18.03.2021	3986	59	NON-PREGNANT	26	3.24	3.49	4.72	3.3
2	25.03.2021	3986	66	INSEMINATION 1	26	2.75	3.77	4.49	3.4
3	05.04.2021	3986	77	INSEMINATION 1	26	3.27	3.51	4.59	3.8
4	12.04.2021	3986	84	INSEMINATION 2	40	3.61	3.15	4.81	3.95
5	19.04.2021	3986	91	INSEMINATION 2	40	3.33	3.47	4.66	3.7
6	26.04.2021	3986	98	INSEMINATION 2	40	2.94	3.66	4.57	3.4
7	05.05.2021	3986	107	INSEMINATION 3	40	3.23	3.32	4.66	4.04
8	19.05.2021	3986		INSEMINATION 3					
9	26.05.2021	3986	121	INSEMINATION 3	51	3.3	3.41	4.7	4.43

n=8

week	DATE	cow number	DIM (days in milk)	state	SCC (10^3/mL)	% Fat	Protein %	Lactose %	MFG Average diameter
1	24.6.20	3871	45	NON- PREGNANT	35	3.07	3.14	5.03	4.06
2	1.7.20	3871	52	NON- PREGNANT	35	3.1	3.12	5.06	3.76
3				NON- PREGNANT					
4	15.7.20	3871	66	NON- PREGNANT	58	3.15	2.99	5.08	4.24
5	21.7.20	3871	72	INSEMINATION 1	58	3.01	3.01	5.06	4.6
6	27.7.20	3871	78	INSEMINATION 1	58	3.05	3.02	4.96	3.95
7	05.08.20	3871	87	INSEMINATION 1	58	3.23	3.39	4.9	4.04
8	12.08.20	3871	94	INSEMINATION 1	92	2.93	3.02	4.86	3.96

9	19.08.20	3871	101	INSEMINATION 1	92	3.2	3.11	5	4.11
10	26.08.20	3871	108	INSEMINATION 1	92	2.53	2.92	5.14	3.59
11	02.09.20	3871	115	INSEMINATION 1	92	3.26	3.12	4.86	4
12	09.09.20	3871	122	INSEMINATION 1	92	3.51	3.08	5.04	4.29
13	16.09.20	3871	129	INSEMINATION 2	140	2.89	3.01	5.01	3.75
14				INSEMINATION 2					
15	1.10.20	3871	144	INSEMINATION 2	140	3.28	3.2	5.04	3.49
16	7.10.20	3871	150	INSEMINATION 2	140	3.38	3.6	4.6	3.88
17	14.10.20	3871	157	INSEMINATION 2	140	3.28	3.25	5.01	3.3
18	22.10.20	3871	165	INSEMINATION 2	145	3.38	3.15	5.02	3.46
19	28.10.20	3871	171	INSEMINATION 2	145	3.2	3.12	4.87	3.2
20	4.11.20	3871	178	INSEMINATION 3/ PREGNANT	145	3.39	3.14	4.98	3.69
21	11.11.2020	3871	185	INSEMINATION 3/ PREGNANT	219	3.1	3.18	4.95	3.78
22	26.11.2020	3871	200	INSEMINATION 3/ PREGNANT	219	3.25	3.23	4.87	3.7
23	03.12.2020	3871	207	INSEMINATION 3/ PREGNANT	219	3.09	3.52	4.8	3.7
24	09.12.2020	3871	213	INSEMINATION 3/ PREGNANT	219	1.62	3.05	5.03	3.96
25	16.12.2020	3871	220	INSEMINATION 3/ PREGNANT	281	1.83	3.18	4.95	3.7

n = 9

week	DATE	cow number	DIM (days in milk)	state	SCC (10^3/mL)	% Fat	Protein %	Lactose %	MFG Average diameter
1	24.6.20	3943	49	NON-PREGNANT	82	3.46	3.37	5.14	3.41
2	1.7.20	3943	56	NON-PREGNANT	82	3.05	3.56	5.08	3.89
3				NON-PREGNANT					
4	15.7.20	3943	70	INSEMINATION 1	53	3.39	3.39	5.11	3.66

5	21.7.20	3943	76	INSEMINATION 1	53	3.44	3.41	5.1	3.85
6	27.7.20	3943	82	INSEMINATION 1	53	3.58	3.37	5.09	3.59
7	05.08.20	3943	91	INSEMINATION 2	53	3.63	3.49	4.94	3.66
8	12.08.20	3943	98	INSEMINATION 2	55	3.16	3.36	4.95	3.6
9	19.08.20	3943	105	INSEMINATION 2	55	3.54	3.4	5.03	3.8
10	26.08.20	3943	112	INSEMINATION 2	55	3.97	3.45	4.82	4.38
11	02.09.20	3943	119	INSEMINATION 2	55	3.95	3.3	4.96	3.2
12	09.09.20	3943	126	INSEMINATION 3	55	4.16	3.49	4.9	3.46
13	16.09.20	3943	133	INSEMINATION 3	67	3.56	3.23	4.96	2.99
14				INSEMINATION 3					
15	1.10.20	3943	148	INSEMINATION 4	67	3.39	3.18	4.93	3.76
16	7.10.20	3943	154	INSEMINATION 4	67	3.8	3.41	4.84	3.8
17	14.10.20	3943	161	INSEMINATION 4	67	3.89	3.34	4.92	3.41
18	22.10.20	3943	169	INSEMINATION 4	57	3.83	3.29	4.94	3.54
19	28.10.20	3943	175	INSEMINATION 4	57	3.59	3.26	4.89	3.03
20	4.11.20	3943	182	INSEMINATION 4	57	3.99	3.32	4.98	3.26
21	11.11.2020	3943	189	INSEMINATION 4	109	3.5	3.36	4.58	2.94
22	26.11.2020	3943	204	INSEMINATION 4	109	3.74	3.77	4.73	3.34
23	3.12.2020	3943	211	INSEMINATION 5	109	4.17	3.67	4.73	3.5
24	09.12.2020	3943	217	INSEMINATION 5	109	3.97	3.45	4.97	3.74
25	16.12.2020	3943	224	INSEMINATION 5	90	4.03	3.72	4.93	3.74
26	23.12.2020	3943	231	INSEMINATION 6/PREGNANT	90	3.97	3.42	4.97	3.56
27	30_12_2020	3943	238	INSEMINATION 6/PREGNANT	90	3.48	3.53	4.51	3.09
28	06.1.2021	3943	245	INSEMINATION 6/PREGNANT	90	3.7	3.47	4.81	
29	13.1.2021	3943	252	INSEMINATION 6/PREGNANT	272	3.12	3.58	4.37	3.04
30	27.1.2021	3943	266	INSEMINATION 6/PREGNANT	272	4.42	3.54	4.97	3.6
31	04.02.2021	3943	274	INSEMINATION 6/PREGNANT	272	3.32	3.41	4.72	3.09

n = 10

week	DATE	cow number	DIM (days in milk)	state	SCC (10^3/mL)	% Fat	Protein %	Lactose %	MFG Average diameter
1	26.11.2020	3509	42	NON-PREGNANT	89	3.16	3.23	4.82	4
2	03.12.2020	3509	49	NON-PREGNANT	89	3.13	3.54	4.67	4.68
3	09.12.2020	3509	55	NON-PREGNANT	89	2.77	3.47	4.47	4.71
4	16.12.2020	3509	62	NON-PREGNANT	55	2.62	3.44	4.9	4.31
5	23.12.2020	3509	69	INSEMINATION 1	55	3.49	3.32	4.89	3.7
6	06.1.2021	3509	83	INSEMINATION 1	55	1.59	3.06	5	3.6
7	13.1.2021	3509	90	INSEMINATION 1	64	2.8	3.21	4.9	3.5
8	27.1.2021	3509	104	INSEMINATION 1	64	2.99	3.4	4.47	4.23
9	04.02.2021	3509	112	INSEMINATION 2/PREGNANT	63	2.05	3.04	4.96	3.59
10	11.02.2021	3509	119	INSEMINATION 2/PREGNANT	64	3.24	3.27	4.91	4.06
11	17.02.2021	3509	125	INSEMINATION 2/PREGNANT	40	3.41	3.19	4.84	3.7
12	23.02.2021	3509	131	INSEMINATION 2/PREGNANT	40	3.82	3.42	4.89	3.2
13	04.03.2021	3509	140	INSEMINATION 2/PREGNANT	40	3.28	3.32	4.79	4.4
14	11.03.2021	3509		INSEMINATION 2/PREGNANT					
15	18.03.2021	3509	154	INSEMINATION 2/PREGNANT	61	3.67	3.47	4.83	3.19
16	25.03.2021	3509	161	INSEMINATION 2/PREGNANT	61	2.92	3.46	4.19	3.59

n = 11

week	DATE	cow number	DIM (days in milk)	state	SCC (10^3/mL)	Fat %	Protein %	Lactose %	MFG Average diameter
1	26.11.2020	4082	63	NON-PREGNANT	103	3.46	3.65	4.92	3.85
2	03.12.2020	4082	70	NON-PREGNANT	103	3.92	3.54	4.89	3.54
3	09.12.2020	4082	76	NON-PREGNANT	103	3.93	3.44	4.77	4.4
4	16.12.2020	4082	83	NON-PREGNANT	216	3.8	3.63	4.78	3.62
5	23.12.2020	4082	90	NON-PREGNANT	213	3.79	3.29	5.04	4.3
6	06.1.2021	4082	104	NON-PREGNANT	213	3.96	3.37	4.94	3.86
7	13.1.2021	4082	111	INSEMINATION 1/PREGNANT	127	3.85	3.29	4.92	3.78
8	27.1.2021	4082	125	INSEMINATION 1/PREGNANT	127	4.11	3.5	4.86	4.06
9	04.02.2021	4082	133	INSEMINATION 1/PREGNANT	127	4.03	3.42	4.83	3.87
10	11.02.2021	4082	140	INSEMINATION 1/PREGNANT	127	3.83	3.39	4.78	3.49
11	17.02.2021	4082	146	INSEMINATION 1/PREGNANT	47	2.65	3.65	4.34	3.85
12	23.02.2021	4082	152	INSEMINATION 1/PREGNANT	47	4.11	3.44	4.77	3.7
13	04.03.2021	4082	161	INSEMINATION 1/PREGNANT	47	4.07	3.16	4.83	3.68

n = 12

week	DATE	cow number	DIM (days in milk)	STATE	SCC (10^3/mL)	Fat %	Protein %	Lactose %	MFG Average diameter
1	26.11.2020	3729	52	NON-PREGNANT	28	2.93	3.26	4.82	4.2
2	03.12.2020	3729	59	NON-PREGNANT	28	2.77	3.54	4.43	4.23
3	09.12.2020	3729	65	NON-PREGNANT	28	3.85	3.03	4.87	4.47
4	16.12.2020	3729	72	NON-PREGNANT	23	2.78	3.56	4.65	4.3
5	23.12.2020	3729	79	NON-PREGNANT	23	3.16	3.2	4.7	5.1
6	06.1.2021	3729	93	INSEMINATION 1	23	2.62	3.3	4.61	3.7
7	13.1.2021	3729	100	INSEMINATION 1	22	3.38	3.36	4.7	4.82
8	27.1.2021	3729	114	INSEMINATION 2	22	2.89	3.22	4.61	4.1
9	04.02.2021	3729	122	INSEMINATION 2	22	2.96	3.16	4.78	3.79
10	11.02.2021	3729	129	INSEMINATION 3	22	3.46	3.18	4.79	3.34
11	17.02.2021	3729	135	INSEMINATION 3	34	2.85	3.37	4.52	3.6
12	23.02.2021	3729	141	INSEMINATION 4	34	3.38	3.3	4.7	3.7
13	04.03.2021	3729	150	INSEMINATION 4	34	2.89	3.17	4.68	4.11
14	11.03.2021	3729		INSEMINATION 4					
15	18.03.2021	3729	164	INSEMINATION 5/PREGNANT	34	2.94	3.15	4.67	4.6
16	25.03.2021	3729	171	INSEMINATION 5/PREGNANT	34	2.9	3.71	4.67	4.16
17	05.04.2021	3729	182	INSEMINATION 5/PREGNANT	34	3.03	3.27	4.38	4.94
18	12.04.2021	3729	189	INSEMINATION 5/PREGNANT	62	3.09	3.17	4.57	3.8
19	19.04.2021	3729	196	INSEMINATION 5/PREGNANT	62	3.22	3.22	4.7	4
20	26.04.2021	3729	203	INSEMINATION 5/PREGNANT	62	3.52	3.45	4.66	4

Table S1B: Microwave Dielectric fitting parameters (Cole-Cole: dielectric strength- $\Delta\varepsilon$, relaxation time τ(s), broadening parameter-α, and conductivity-σ(Siemens/m) measured **weekly** for n = 12 different cows at different reproductive states (Non-pregnant-NP, during inseminations and during confirmed pregnancy). The not measured days correspond mostly to Saturdays an official holiday in Israel.

n = 1

week	DATE	cow number	STATE	Δε	τ (s)	α	σ(S/m)
1	13.5.20	4027	NON-PREGNANT	60.90	8.48E-12	0.98	0.51
2	20.5.20	4027	NON-PREGNANT	56.93	8.69E-12	0.98	0.45
3	27.5.20	4027	NON-PREGNANT	61.28	8.58E-12	0.97	0.50
4	3.6.20	4027	NON-PREGNANT	60.22	8.71E-12	0.98	0.51
5	10.6.20	4027	NON-PREGNANT	61.12	9.13E-12	0.97	0.52
6	17.6.20	4027	NON-PREGNANT	59.93	9.02E-12	0.99	0.50
7	24.6.20	4027	INSEMINATION 1	60.36	8.70E-12	0.98	0.47
8	1.7.20	4027	INSEMINATION 1	58.54	8.87E-12	0.98	0.51
9			INSEMINATION 1				
10	15.7.20	4027	INSEMINATION 1	58.11	9.13E-12	1.00	0.49
11	21.7.20	4027	INSEMINATION 1	61.29	8.72E-12	0.97	0.50
12	27.7.20	4027	INSEMINATION 1	59.87	8.75E-12	0.98	0.49
13	05.08.20	4027	INSEMINATION 1	60.93	8.85E-12	0.97	0.50
14	12.08.20	4027	INSEMINATION 1				
15	19.08.20	4027	INSEMINATION 1	58.81	9.17E-12	0.97	0.50
16	26.08.20	4027	INSEMINATION 2				
17	02.09.20	4027	INSEMINATION 2	59.76	9.05E-12	0.98	0.49
18	09.09.20	4027	INSEMINATION 2	58.64	8.93E-12	0.97	0.51
19	16.09.20	4027	INSEMINATION 2	58.55	9.13E-12	0.99	0.52
20			INSEMINATION 2				

21	1.10.20	4027	INSEMINATION 2	57.03	8.75E-12	1.00	0.46
22	7.10.20	4027	INSEMINATION 2	55.95	9.01E-12	1.00	0.46
23	14.10.20	4027	INSEMINATION 2	58.35	8.81E-12	0.99	0.50
24	22.10.20	4027	INSEMINATION 2	57.45	8.78E-12	0.99	0.47
25	28.10.20	4027	INSEMINATION 2	57.58	8.93E-12	0.99	0.47
26	04.11.20	4027	INSEMINATION 3	56.09	9.10E-12	1.00	0.47
27	11.11.2020	4027	INSEMINATION 3	58.68	8.72E-12	0.98	0.45
28	26.11.2020	4027	INSEMINATION 3	59.68	8.74E-12	1.00	0.42
29	3.12.2020	4027	INSEMINATION 3	60.18	8.58E-112	1.00	0.41
30	09.12.2020	4027	INSEMINATION 4	58.29	8.63E-12	0.98	0.47
31	16.12.2020	4027	INSEMINATION 4	60.19	8.97E-12	0.97	0.44
32	23.12.2020	4027	INSEMINATION 4	56.92	8.99E-12	0.98	0.42
33	30.12.2020	4027	INSEMINATION 5	60.05	8.89E-12	0.98	0.45
34	06.1.2021	4027	INSEMINATION 5	58.29	9.09E-12	0.98	0.45
35	13.1.2021	4027	INSEMINATION 5	50.32	8.72E-12	0.98	0.37
36	27.1.2021	4027	INSEMINATION 5	55.53	8.57E-12	0.98	0.39
37	04.02.2021	4027	INSEMINATION 6	55.39	8.74E-12	0.98	0.42
38	11.02.2021	4027	INSEMINATION 6	58.04	8.62E-12	0.98	0.42
39	17.02.2021	4027	INSEMINATION 6	58.16	8.59E-12	0.98	0.44
40	23.02.2021	4027	INSEMINATION 6	55.08	8.66E-12	0.98	0.40
41	04.03.2021	4027	INSEMINATION 6	54.21	8.54E-12	0.98	0.41
42	11.03.2021	4027	INSEMINATION 6				
43	18.03.2021	4027	INSEMINATION 6	48.38	8.8E-12	0.98	0.39
44	25.03.2021	4027	INSEMINATION 6	56.40	8.72E-12	0.98	0.43
45	05.04.2021	4027	INSEMINATION 6	53.68	8.92E-12	0.98	0.40
46	12.04.2021	4027	INSEMINATION 6	55.23	8.80E-12	0.98	0.45
47	19.04.2021	4027	INSEMINATION 7	53.95	9.00E-12	0.98	0.44

48	26.04.2021	4027	INSEMINATION 7	43.89	8.72E-12	0.98	0.35
49	05.05.2021	4027	INSEMINATION 7	55.92	9.21E-12	0.98	0.43
50	19.05.2021	4027	INSEMINATION 7	54.73	9.07E-12	0.98	0.41
51	26.05.2021	4027	INSEMINATION 7	55.52	9.03E-12	0.98	0.43

n = 2

week	DATE	cow number	STATE	Δε	τ (s)	α	σ(S/m)
1	13.5.20	4032	NON-PREGNANT	56.95	8.48E-12	0.98	0.41
2	20.5.20	4032	NON-PREGNANT	56.00	8.82E-12	1.00	0.39
3	27.5.20	4032	NON-PREGNANT	55.73	8.69E-12	1.00	0.30
4	3.6.20	4032	NON-PREGNANT	57.82	8.82E-12	0.99	0.41
5	10.6.20	4032	NON-PREGNANT	58.91	9.18E-12	0.97	0.44
6	17.6.20	4032	NON-PREGNANT	59.60	9.03E-12	0.99	0.42
7	24.6.20	4032	INSEMINATION 1	59.27	8.74E-12	0.98	0.41
8	1.7.20	4032	INSEMINATION 1	56.75	9.03E-12	1.00	0.44
9			INSEMINATION 1				
10	15.7.20	4032	INSEMINATION 1	57.08	9.25E-12	1.00	0.45
11	21.7.20	4032	INSEMINATION 1	59.56	8.76E-12	0.98	0.44
12	27.7.20	4032	INSEMINATION 1	58.45	9.18E-12	0.99	0.46
13	05.08.20	4032	INSEMINATION 1	59.36	8.93E-12	0.97	0.44
14	12.08.20	4032	INSEMINATION 1	58.62	9.11E-12	0.98	0.45
15	19.08.20	4032	INSEMINATION 1	57.77	9.35E-12	0.97	0.44
16	26.08.20	4032	INSEMINATION 2				
17	02.09.20	4032	INSEMINATION 2	57.07	9.06E-12	0.99	0.42
18	09.09.20	4032	INSEMINATION 2	56.33	8.97E-12	0.98	0.43

19	16.09.20	4032	INSEMINATION 2	57.44	9.20E-12	0.99	0.44
20			INSEMINATION 2				
21	1.10.20	4032	INSEMINATION 2	56.59	8.75E-12	1.00	0.40
22	7.10.20	4032	INSEMINATION 2	56.59	9.02E-12	0.99	0.42
23	14.10.20	4032	INSEMINATION 2	57.19	8.88E-12	0.99	0.41
24	22.10.20	4032	INSEMINATION 2	57.15	8.85E-12	0.99	0.41
25	28.10.20	4032	INSEMINATION 2	56.40	8.97E-12	1.00	0.41
26	04.11.20	4032	INSEMINATION 3	55.36	9.17E-12	1.00	0.40
27	11.11.2020	4032	INSEMINATION 3	57.97	8.69E-12	0.98	0.38
28	26.11.2020	4032	INSEMINATION 3	59.35	8.74E-12	1.00	0.36
29	3.12.2020	4032	INSEMINATION 3	58.58	8.49E-12	1.00	0.37
30	09.12.2020	4032	INSEMINATION 4	59.61	8.73E-12	0.98	0.41
31	16.12.2020	4032	INSEMINATION 4	54.19	8.96E-12	0.98	0.35
32	23.12.2020	4032	INSEMINATION 4	60.55	8.83E-12	0.96	0.34
33	30.12.2021	4032	INSEMINATION 5	54.93	8.93E-12	0.98	0.36
34	06.1.2021	4032	INSEMINATION 5	55.36	9.00E-12	0.99	0.36
35	13.1.2021	4032	INSEMINATION 5	55.66	8.73E-12	0.98	0.37
36	27.1.2021	4032	INSEMINATION 5	57.09	8.64E-12	0.98	0.36
37	04.02.2021	4032	INSEMINATION 6	56.72	8.89E-12	0.98	0.38
38	11.02.2021	4032	INSEMINATION 6	53.24	8.60E-12	0.98	0.36
39	17.02.2021	4032	INSEMINATION 6	56.64	8.69E-12	0.98	0.37
40	23.02.2021	4032	INSEMINATION 6	56.62	8.65E-12	0.98	0.37
41	04.03.2021	4032	INSEMINATION 6	57.48	8.65E-12	0.98	0.39
42	11.03.2021	4032	INSEMINATION 6				
43	18.03.2021	4032	INSEMINATION 6	51.76	8.81E-12	0.98	0.38
44	25.03.2021	4032	INSEMINATION 6	57.84	8.75E-12	0.98	0.41
45	05.04.2021	4032	INSEMINATION 6	57.26	8.89E-12	0.98	0.37

46	12.04.2021	4032	INSEMINATION 6				
47	19.04.2021	4032	INSEMINATION 7	52.09	9.19E-12	0.98	0.35
48	26.04.2021	4032	INSEMINATION 7	47.99	8.84E-12	0.98	0.33
49	05.05.2021	4032	INSEMINATION 7	57.10	9.19E-12	0.97	0.40
50	19.05.2021	4032	INSEMINATION 7	58.06	9.05E-12	0.98	0.40
51	26.05.2021	4032	INSEMINATION 7	56.29	9.09E-12	0.98	0.38

n = 3

week	DATE	cow number	STATE	Δε	τ (s)	α	σ(S/m)
1	26.11.2020	3802	NON-PREGNANT	52.16	8.50E-12	1.00	0.38
2	03.12.2020	3802	NON-PREGNANT	56.30	8.3E-12	1.00	0.36
3	09.12.2020	3802	NON-PREGNANT	60.81	8.52E-12	0.98	0.44
4	16.12.2020	3802	NON-PREGNANT	58.67	8.96E-12	0.98	0.41
5	23.12.2020	3802	NON-PREGNANT	63.78	9.23E-12	0.98	0.44
6	30.12.2020	3802	INSEMINATION 1	52.64	8.68E-12	0.98	0.39
7	06.1.2021	3802	INSEMINATION 1	59.98	9.01E-12	0.99	0.44
8	13.1.2021	3802	INSEMINATION 1	60.15	8.71E-12	0.98	0.44
9	27.1.2021	3802	INSEMINATION 2	57.75	8.54E-12	0.98	0.42
10	04.02.2021	3802	INSEMINATION 2	56.10	8.9E-12	0.98	0.43
11	11.02.2021	3802	INSEMINATION 2	49.71	8.52E-12	0.98	0.34
12	17.02.2021	3802	INSEMINATION 2	56.76	8.45E-12	0.98	0.48
13	23.02.2021	3802	INSEMINATION 2	58.86	8.53E-12	0.98	0.42
14	04.03.2021	3802	INSEMINATION 3	58.18	8.62E-12	0.98	0.44
15	11.03.2021	3802	INSEMINATION 3				
16	18.03.2021	3802	INSEMINATION 3	52.76	8.88E-12	0.98	0.42

17	25.03.2021	3802	INSEMINATION 3	60.17	8.70E-12	0.98	0.47
18	05.04.2021	3802	INSEMINATION 4	60.09	8.63E-12	0.98	0.46
19	12.04.2021	3802	INSEMINATION 4	57.38	8.76E-12	0.98	0.42
20	19.04.2021	3802	INSEMINATION 5	55.00	9.06E-12	0.98	0.45
21	26.04.2021	3802	INSEMINATION 5	49.30	8.67E-12	0.98	0.38
22	05.05.2021	3802	INSEMINATION 5	57.76	9.31E-12	0.98	0.47
23	19.05.2021	3802	INSEMINATION 6	60.53	9.01E-12	0.98	0.45
24	26.05.2021	3802	INSEMINATION 6	55.02	8.93E-12	0.98	0.44

n = 4

week	DATE	cow number	STATE	Δε	τ (s)	α	σ(S/m)
1	23.12.2020	3971	NON-PREGNANT	61.12	8.79E-12	0.97	0.42
2	06.1.2021	3971	NON-PREGNANT	58.10	9.02E-12	0.99	0.44
3	13.1.2021	3971	NON-PREGNANT	58.59	8.84E-12	0.98	0.43
4	27.1.2021	3971	NON-PREGNANT	55.64	8.50E-12	0.98	0.40
5	04.02.2021	3971	NON-PREGNANT	59.59	8.80E-12	0.98	0.45
6	11.02.2021	3971	NON-PREGNANT	60.96	8.63E-12	0.98	0.46
7	17.02.2021	3971	NON-PREGNANT	58.39	8.56E-12	0.98	0.43
8	23.02.2021	3971	NON-PREGNANT	59.39	8.53E-12	0.98	0.43
9	04.03.2021	3971	INSEMINATION 1	59.41	8.61E-12	0.98	0.45
10	11.03.2021	3971	INSEMINATION 1				
11	18.03.2021	3971	INSEMINATION 1	59.14	8.86E-12	0.98	0.44
12	25.03.2021	3971	INSEMINATION 1	60.05	9.01E-12	0.98	0.44
13	05.04.2021	3971	INSEMINATION 2	57.36	8.79E-12	0.98	0.41
14	12.04.2021	3971	INSEMINATION 2	59.34	8.77E-12	0.98	0.40

15	19.04.2021	3971	INSEMINATION 2	57.43	9.08E-12	0.98	0.44
16	26.04.2021	3971	INSEMINATION 2	48.06	8.65E-12	0.98	0.37
17	05.05.2021	3971	INSEMINATION 2	57.74	8.95E-12	0.98	0.42
18	19.05.2021	3971	INSEMINATION 2	59.88	9.04E-12	0.98	0.44
19	26.05.2021	3971	INSEMINATION 3	57.74	8.95E-12	0.98	0.42

n= 5

week	DATE	cow number	STATE	Δε	τ (s)	α	σ(S/m)
1	04.02.2021	3915	NON-PREGNANT	57.41	8.79E-12	0.99	0.47
2	11.02.2021	3915	NON-PREGNANT	58.74	8.50E-12	0.98	0.49
3	17.02.2021	3915	NON-PREGNANT	58.68	8.42E-12	0.98	0.47
4	23.02.2021	3915	NON-PREGNANT	60.11	8.43E-12	0.98	0.49
5	04.03.2021	3915	INSEMINATION 1	58.44	8.52E-12	0.98	0.49
6	11.03.2021	3915	INSEMINATION 1				
7	18.03.2021	3915	INSEMINATION 1	57.19	8.82E-12	0.98	0.50
8	25.03.2021	3915	INSEMINATION 1	58.55	8.94E-12	0.98	0.48
9	05.04.2021	3915	INSEMINATION 1	60.34	8.80E-12	0.98	0.49
10	12.04.2021	3915	INSEMINATION 1				
11	19.04.2021	3915	INSEMINATION 1	60.32	9.13E-12	0.98	0.52
12	26.04.2021	3915	INSEMINATION 1	51.32	8.67E-12	0.98	0.40
13	05.05.2021	3915	INSEMINATION 2	57.14	9.34E-12	0.98	0.47
14	19.05.2021	3915	INSEMINATION 2	61.62	9.00E-12	0.98	0.49
15	26.05.2021	3915	INSEMINATION 2	59.25	8.97E-12	0.98	0.45

n = 6

week	DATE	cow number	STATE	Δε	τ (s)	α	σ(S/m)
1	18.03.2021	3991	NON-PREGNANT	49.27	8.88E-12	0.98	0.39
2	25.03.2021	3991	NON-PREGNANT	61.05	8.92E-12	0.98	0.46
3	05.04.2021	3991	NON-PREGNANT	58.42	8.73E-12	0.98	0.42
4	12.04.2021	3991	NON-PREGNANT	60.13	8.78E-12	0.98	0.42
5	19.04.2021	3991	NON-PREGNANT	58.86	9.13E-12	0.97	0.44
6	26.04.2021	3991	INSEMINATION 1	49.52	8.75E-12	0.98	0.35
7	05.05.2021	3991	INSEMINATION 1	57.63	9.56E-12	0.98	0.43
8	19.05.2021	3991	INSEMINATION 1	61.54	9.03E-12	0.98	0.45
9	26.05.2021	3991	INSEMINATION 2	57.63	8.89E-12	0.98	0.42

n = 7

week	DATE	cow number	STATE	Δε	τ (s)	α	σ(S/m)
1	18.03.2021	3986	NON-PREGNANT	59.48	8.88E-12	0.98	0.49
2	25.03.2021	3986	INSEMINATION 1	57.90	8.84E-12	0.98	0.45
3	05.04.2021	3986	INSEMINATION 1	60.94	8.79E-12	0.98	0.47
4	12.04.2021	3986	INSEMINATION 2	59.52	8.69E-12	0.98	0.44
5	19.04.2021	3986	INSEMINATION 2	59.10	9.09E-12	0.98	0.49
6	26.04.2021	3986	INSEMINATION 2	50.69	8.81E-12	0.98	0.41
7	05.05.2021	3986	INSEMINATION 3	59.69	9.52E-12	0.98	0.50
8	19.05.2021	3986	INSEMINATION 3	53.28	8.98E-12	0.98	0.43
9	26.05.2021	3986	INSEMINATION 3	62.25	8.96E-12	0.98	0.50

n = 8

week	DATE	cow number	STATE	Δε	τ (s)	α	σ(S/m)
1	24.6.20	3871	NON- PREGNANT	58.33	8.73E-12	0.99	0.46
2	1.7.20	3871	NON- PREGNANT	60.77	8.68E-12	0.98	0.46
3			NON- PREGNANT				
4	15.7.20	3871	NON- PREGNANT	59.43	9.16E-12	1.00	0.51
5	21.7.20	3871	INSEMINATION 1	58.65	8.78E-12	0.98	0.46
6	27.7.20	3871	INSEMINATION 1	61.49	9.05E-12	0.98	0.51
7	05.08.20	3871	INSEMINATION 1	59.36	8.94E-12	0.98	0.49
8	12.08.20	3871	INSEMINATION 1	59.66	9.09E-12	0.99	0.50
9	19.08.20	3871	INSEMINATION 1	56.94	9.38E-12	0.97	0.52
10	26.08.20	3871	INSEMINATION 1				
11	02.09.20	3871	INSEMINATION 1	58.45	8.96E-12	0.98	0.47
12	09.09.20	3871	INSEMINATION 1	59.55	8.97E-12	0.98	0.50
13	16.09.20	3871	INSEMINATION 2	59.39	9.1E-12	0.99	0.50
14			INSEMINATION 2				
15	1.10.20	3871	INSEMINATION 2	57.41	8.77E-12	1.00	0.46
16	7.10.20	3871	INSEMINATION 2	56.02	8.82E-12	0.99	0.49
17	14.10.20	3871	INSEMINATION 2	57.25	8.81E-12	0.99	0.46
18	22.10.20	3871	INSEMINATION 2	56.64	8.83E-12	0.99	0.46
19	28.10.20	3871	INSEMINATION 2	58.07	8.96E-12	0.99	0.50
20	4.11.20	3871	INSEMINATION 3/ PREGNANT	57.77	8.95E-12	1.00	0.45
21	11.11.2020	3871	INSEMINATION 3/ PREGNANT	59.66	8.7E-12	0.98	0.43
22	26.11.2020	3871	INSEMINATION 3/ PREGNANT	61.95	8.69E-12	1.00	0.53

23	03.12.2020	3871	INSEMINATION 3/ PREGNANT	58.69	8.49E-12	1.00	0.48
24	09.12.2020	3871	INSEMINATION 3/ PREGNANT	58.18	8.68E-12	0.99	0.49
25	16.12.2020	3871	INSEMINATION 3/ PREGNANT	55.22	8.85E-12	0.98	0.41

n = 9

week	DATE	cow number	STATE	Δε	τ (s)	α	σ(S/m)
1	24.6.20	3943	NON-PREGNANT	59.55	8.80E-12	0.99	0.48
2	1.7.20	3943	NON-PREGNANT	59.92	8.82E-12	0.99	0.48
3			NON-PREGNANT				
4	15.7.20	3943	INSEMINATION 1	57.96	9.22E-12	1.00	0.48
5	21.7.20	3943	INSEMINATION 1	59.45	8.78E-12	0.99	0.51
6	27.7.20	3943	INSEMINATION 1	59.35	9.14E-12	0.98	0.51
7	05.08.20	3943	INSEMINATION 2	58.74	9.00E-12	0.98	0.47
8	12.08.20	3943	INSEMINATION 2	58.50	9.06E-12	0.99	0.51
9	19.08.20	3943	INSEMINATION 2	58.28	9.09E-12	0.97	0.51
10	26.08.20	3943	INSEMINATION 2				
11	02.09.20	3943	INSEMINATION 2	59.73	9.03E-12	0.98	0.47
12	09.09.20	3943	INSEMINATION 3	58.65	8.90E-12	0.98	0.50
13	16.09.20	3943	INSEMINATION 3	58.72	9.07E-12	0.98	0.49
14			INSEMINATION 3				
15	1.10.20	3943	INSEMINATION 4	57.59	8.76E-12	1.00	0.44
16	7.10.20	3943	INSEMINATION 4	56.81	8.93E-12	0.99	0.47
17	14.10.20	3943	INSEMINATION 4	57.70	8.82E-12	0.99	0.47
18	22.10.20	3943	INSEMINATION 4	56.88	8.89E-12	0.99	0.47
19	28.10.20	3943	INSEMINATION 4	58.04	8.95E-12	0.99	0.47

20	4.11.20	3943	INSEMINATION 4	56.35	8.98E-12	1.00	0.47
21	11.11.2020	3943	INSEMINATION 4	59.26	8.69E-12	0.98	0.45
22	26.11.2020	3943	INSEMINATION 4	63.67	8.71E-12	0.99	0.47
23	3.12.2020	3943	INSEMINATION 5	57.62	8.45E-12	1.00	0.43
24	09.12.2020	3943	INSEMINATION 5	57.86	8.61E-12	0.98	0.47
25	16.12.2020	3943	INSEMINATION 5	58.50	8.93E-12	0.98	0.44
26	23.12.2020	3943	INSEMINATION 6/PREGNANT	57.25	8.85E-12	0.98	0.43
27	30.12.2020	3943	INSEMINATION 6/PREGNANT	54.03	8.60E-12	0.98	0.43
28	06.1.2021	3943	INSEMINATION 6/PREGNANT				
29	13.1.2021	3943	INSEMINATION 6/PREGNANT	54.09	8.65E-12	0.98	0.44
30	27.1.2021	3943	INSEMINATION 6/PREGNANT	55.21	8.62E-12	0.99	0.40
31	04.02.2021	3943	INSEMINATION 6/PREGNANT	55.64	8.76E-12	0.98	0.43

n = 10

week	DATE	cow number	STATE	Δε	τ (s)	α	σ(S/m)
1	26.11.2020	3509	NON-PREGNANT	60.41	8.71E-12	1.00	0.44
2	03.12.2020	3509	NON-PREGNANT	60.29	8.58E-12	1.00	0.42
3	09.12.2020	3509	NON-PREGNANT	59.20	8.63E-12	0.98	0.50
4	16.12.2020	3509	NON-PREGNANT	64.01	8.82E-12	0.97	0.48
5	23.12.2020	3509	INSEMINATION 1	59.26	8.83E-12	0.98	0.46
6	06.1.2021	3509	INSEMINATION 1	59.59	9.01E-12	0.98	0.48
7	13.1.2021	3509	INSEMINATION 1	61.06	8.66E-12	0.98	0.48
8	27.1.2021	3509	INSEMINATION 1	53.40	8.43E-12	0.98	0.38

9	04.02.2021	3509	INSEMINATION 2/PREGNANT	56.20	8.80E-12	0.98	0.43
10	11.02.2021	3509	INSEMINATION 2/PREGNANT	60.95	8.61E-12	0.98	0.48
11	17.02.2021	3509	INSEMINATION 2/PREGNANT	58.98	8.56E-12	0.98	0.44
12	23.02.2021	3509	INSEMINATION 2/PREGNANT	57.99	8.62E-12	0.98	0.45
13	04.03.2021	3509	INSEMINATION 2/PREGNANT	59.43	8.55E-12	0.98	0.46
14	11.03.2021	3509	INSEMINATION 2/PREGNANT				
15	18.03.2021	3509	INSEMINATION 2/PREGNANT	55.92	8.74E-12	0.98	0.45
16	25.03.2021	3509	INSEMINATION 2/PREGNANT	59.63	8.76E-12	0.98	0.45

n = 11

week	DATE	cow number	STATE	Δε	τ (s)	α	σ(S/m)
1	26.11.2020	4082	NON-PREGNANT	60.33	8.73E-12	1.00	0.40
2	03.12.2020	4082	NON-PREGNANT	58.01	8.59E-12	1.00	0.39
3	09.12.2020	4082	NON-PREGNANT	60.24	8.61E-12	0.98	0.45
4	16.12.2020	4082	NON-PREGNANT	60.06	8.96E-12	0.98	0.43
5	23.12.2020	4082	NON-PREGNANT	56.98	8.89E-12	0.98	0.46
6	06.1.2021	4082	NON-PREGNANT	58.99	8.96E-12	0.98	0.45
7	13.1.2021	4082	INSEMINATION 1/PREGNANT	52.74	8.63E-12	0.99	0.41
8	27.1.2021	4082	INSEMINATION 1/PREGNANT	55.92	8.62E-12	0.98	0.41
9	04.02.2021	4082	INSEMINATION 1/PREGNANT				
10	11.02.2021	4082	INSEMINATION 1/PREGNANT	59.08	8.62E-12	0.98	0.45
11	17.02.2021	4082	INSEMINATION 1/PREGNANT	58.67	8.60E-12	0.98	0.45
12	23.02.2021	4082	INSEMINATION 1/PREGNANT	59.77	8.50E-12	0.98	0.44
13	04.03.2021	4082	INSEMINATION 1/PREGNANT	56.42	8.51E-12	0.98	0.42

n = 12

week	DATE	cow number	STATE	Δε	τ (s)	α	σ(S/m)
1	26.11.2020	3729	NON- PREGNANT	62.38	8.72E-12	1.00	0.48
2	03.12.2020	3729	NON- PREGNANT	61.04	8.61E-12	1.00	0.43
3	09.12.2020	3729	NON- PREGNANT	57.21	8.82E-12	0.99	0.49
4	16.12.2020	3729	NON- PREGNANT	61.57	8.89E-12	0.98	0.50
5	23.12.2020	3729	NON- PREGNANT				
6	06.1.2021	3729	INSEMINATION 1	59.31	8.86E-12	1.00	0.50
7	13.1.2021	3729	INSEMINATION 1	52.75	8.66E-12	1.00	0.43
8	27.1.2021	3729	INSEMINATION 2	61.90	8.51E-12	0.98	0.48
9	04.02.2021	3729	INSEMINATION 2	61.53	8.87E-12	0.98	0.52
10	11.02.2021	3729	INSEMINATION 3	56.34	8.50E-12	0.98	0.46
11	17.02.2021	3729	INSEMINATION 3	57.69	8.49E-12	0.98	0.41
12	23.02.2021	3729	INSEMINATION 4	60.58	8.53E-12	0.98	0.51
13	04.03.2021	3729	INSEMINATION 4	57.85	8.49E-12	0.98	0.49
14	11.03.2021	3729	INSEMINATION 4				
15	18.03.2021	3729	INSEMINATION 5/PREGNANT	59.17	8.81E-12	0.97	0.47
16	25.03.2021	3729	INSEMINATION 5/PREGNANT	61.14	8.71E-12	0.98	0.52
17	05.04.2021	3729	INSEMINATION 5/PREGNANT	60.39	8.67E-12	0.98	0.46
18	12.04.2021	3729	INSEMINATION 5/PREGNANT	58.76	8.72E-12	0.98	0.47
19	19.04.2021	3729	INSEMINATION 5/PREGNANT	52.60	8.97E-12	0.98	0.46
20	26.04.2021	3729	INSEMINATION 5/PREGNANT	47.95	8.75E-12	0.98	0.41

SUPPORTING INFORMATION 2: Physiological and Microwave Dielectric fitting parameters measured daily for individual tracked cows among three different reproductive states: Non-Pregnant, insemination, and pregnancy.

Table S2A: Physiological parameters (fat %, density %, lactose%, solid non-fat (SNF%), and protein%) as well as weather temperature on the day of sample collection measured daily for n=10 different cows at different reproductive states (Non-pregnant-NP, Inseminations and during confirmed pregnancy). The not measured days correspond mostly to Saturdays, an official holiday in Israel.

n = 1

Day	cow number	state	weather temperature (Celsius)	FAT%	Density%	Lactose%	SNF%	Protein%
1	4165	NON-PREGNANT	11	3.54	28.64	4.42	8.06	3.25
2	4165	NON-PREGNANT	11	2.15	30.46	4.53	8.24	3.31
3	4165	NON-PREGNANT	10	1.88	30.48	4.5	8.18	3.3
4	4165	NON-PREGNANT	11	2.8	29.7	4.49	8.17	3.29
5	4165	NON-PREGNANT	9	2.32	30.55	4.56	8.29	3.34
6	4165	FIRST INSEMINATION/PREGNANT	8	2.65	30.43	4.57	8.33	3.35
7	4165	FIRST INSEMINATION/PREGNANT						
8	4165	FIRST INSEMINATION/PREGNANT	9	2.1	30.21	4.48	8.16	3.29
9	4165	FIRST INSEMINATION/PREGNANT						
10	4165	FIRST INSEMINATION/PREGNANT	8	2.11	31.24	4.63	8.43	3.38
11	4165	FIRST INSEMINATION/PREGNANT	7	2.16	30.57	4.54	8.27	3.33
12	4165	FIRST INSEMINATION/PREGNANT	10	1.78	31.97	4.7	8.55	3.43
13	4165	FIRST INSEMINATION/PREGNANT	10	1.57	31.87	4.66	8.48	3.4

14	4165	FIRST INSEMINATION/PREGNANT							
15	4165	FIRST INSEMINATION/PREGNANT	6	1.39	30.82	4.49	8.17	3.29
16	4165	FIRST INSEMINATION/PREGNANT	5	2.19	32.14	4.77	8.67	3.48
17	4165	FIRST INSEMINATION/PREGNANT	3	1.78	32.11	4.72	8.58	3.44
18	4165	FIRST INSEMINATION/PREGNANT	6	1.8	31.97	4.7	8.55	3.43
19	4165	FIRST INSEMINATION/PREGNANT	4	2.01	31.87	4.71	8.57	3.44
20	4165	FIRST INSEMINATION/PREGNANT	5	2.26	31.71	4.71	8.58	3.44
21	4165	FIRST INSEMINATION/PREGNANT						
22	4165	FIRST INSEMINATION/PREGNANT						
23	4165	FIRST INSEMINATION/PREGNANT	5	2.26	32.65	4.85	8.82	3.53
24	4165	FIRST INSEMINATION/PREGNANT	4	1.76	33.34	4.89	8.89	3.56
25	4165	FIRST INSEMINATION/PREGNANT	8	2.08	33.26	4.91	8.94	3.57
26	4165	FIRST INSEMINATION/PREGNANT	7	2.54	32.79	4.9	8.91	3.56
27	4165	FIRST INSEMINATION/PREGNANT	8	1.47	32.89	4.79	8.72	3.49
28	4165	FIRST INSEMINATION/PREGNANT						
29	4165	FIRST INSEMINATION/PREGNANT	5	1.92	32.91	4.85	8.81	3.53
30	4165	FIRST INSEMINATION/PREGNANT	7	2.34	31.86	4.74	8.63	3.46
31	4165	FIRST INSEMINATION/PREGNANT	9	2.3	32.48	4.83	8.78	3.51
32	4165	FIRST INSEMINATION/PREGNANT	5	1.66	32.7	4.79	8.71	3.49
33	4165	FIRST INSEMINATION/PREGNANT	6	2.13	32.41	4.8	8.73	3.5
34	4165	FIRST INSEMINATION/PREGNANT	11	2.22	32.82	4.87	8.85	3.54
35	4165	FIRST INSEMINATION/PREGNANT						
36	4165	FIRST INSEMINATION/PREGNANT	10	1.76	27.29	4.03	7.34	2.99
37	4165	FIRST INSEMINATION/PREGNANT	9	2.05	32.61	4.82	8.76	3.51
38	4165	FIRST INSEMINATION/PREGNANT	8	2.31	32.01	4.76	8.66	3.47

39	4165	FIRST INSEMINATION/PREGNANT	7	1.85	33.23	4.88	8.88	3.55
40	4165	FIRST INSEMINATION/PREGNANT	8	2.2	32.07	4.76	8.66	3.47
41	4165	FIRST INSEMINATION/PREGNANT	8	2.62	29.8	4.48	8.16	3.29
42	4165	FIRST INSEMINATION/PREGNANT						
43	4165	FIRST INSEMINATION/PREGNANT	9	2.37	33.07	4.92	8.95	3.58
44	4165	FIRST INSEMINATION/PREGNANT	13	2.36	32.28	4.8	8.74	3.5
45	4165	FIRST INSEMINATION/PREGNANT	11	2.37	32.35	4.81	8.76	3.51
46	4165	FIRST INSEMINATION/PREGNANT	10	2.18	31.72	4.71	8.56	3.43
47	4165	FIRST INSEMINATION/PREGNANT	10	2.11	31.97	4.73	8.61	3.45
48	4165	FIRST INSEMINATION/PREGNANT	8	2.07	31.81	4.71	8.56	3.44
49	4165	FIRST INSEMINATION/PREGNANT						
50	4165	FIRST INSEMINATION/PREGNANT						
51	4165	FIRST INSEMINATION/PREGNANT	13	2.34	32.87	4.88	8.89	3.55
52	4165	FIRST INSEMINATION/PREGNANT	14	2.38	33.42	4.97	9.04	3.61
53	4165	FIRST INSEMINATION/PREGNANT	16	2.48	32.19	4.8	8.75	3.5
54	4165	FIRST INSEMINATION/PREGNANT	13	2.16	32.64	4.83	8.79	3.52
55	4165	FIRST INSEMINATION/PREGNANT	14	2.14	32.48	4.81	8.75	3.5
56	4165	FIRST INSEMINATION/PREGNANT						
57	4165	FIRST INSEMINATION/PREGNANT	10	2.37	32.37	4.82	8.77	3.51
58	4165	FIRST INSEMINATION/PREGNANT	10	1.91	32.61	4.8	8.74	3.5
59	4165	FIRST INSEMINATION/PREGNANT	10	2.29	32.95	4.89	8.9	3.56
60	4165	FIRST INSEMINATION/PREGNANT	12	2.14	32.29	4.78	8.7	3.49
61	4165	FIRST INSEMINATION/PREGNANT	12	1.82	32.34	4.76	8.65	3.47
62	4165	FIRST INSEMINATION/PREGNANT	11	2.02	32.85	4.85	8.82	3.53
63	4165	FIRST INSEMINATION/PREGNANT						

64	4165	FIRST INSEMINATION/PREGNANT	12	2.36	32.44	4.83	8.79	3.52
65	4165	FIRST INSEMINATION/PREGNANT	12	2.29	31.42	4.68	8.51	3.42

n = 2

Day	cow number	state	weather temperature (Celsius)	FAT%	Density%	Lactose%	SNF%	Protein%
1	4151	NON-PREGNANT	11	3.23	30.17	4.6	8.38	3.37
2	4151	NON-PREGNANT	11	2.77	30.99	4.67	8.5	3.41
3	4151	NON-PREGNANT	10	2.33	30.85	4.6	8.37	3.36
4	4151	NON-PREGNANT	11	3.47	31.02	4.75	8.65	3.47
5	4151	NON-PREGNANT	9	3.81	30.38	4.69	8.56	3.43
6	4151	NON-PREGNANT	8	2.58	30.37	4.56	8.3	3.34
7	4151	NON-PREGNANT	10					
8	4151	NON-PREGNANT	9	3.43	29.76	4.56	8.32	3.34
9	4151	NON-PREGNANT	11	3.37	31.65	4.82	8.79	3.52
10	4151	NON-PREGNANT	8	3.66	30.57	4.7	8.57	3.44
11	4151	NON-PREGNANT	7	3.77	31.13	4.79	8.74	3.5
12	4151	NON-PREGNANT	10	2.65	32.35	4.84	8.82	3.53
13	4151	NON-PREGNANT	10	3.02	31.54	4.77	8.69	3.48
14	4151	NON-PREGNANT	9					
15	4151	FIRST INSEMINATION/PREGNANT	6	3.77	29.28	4.53	8.27	3.32
16	4151	FIRST INSEMINATION/PREGNANT	5	4.02	31.59	4.89	8.91	3.56
17	4151	FIRST INSEMINATION/PREGNANT	3	3.15	31.09	4.72	8.6	3.45
18	4151	FIRST INSEMINATION/PREGNANT	6	3.16	31.91	4.84	8.82	3.53
19	4151	FIRST INSEMINATION/PREGNANT	4	3.35	31.98	4.87	8.87	3.55

20	4151	FIRST INSEMINATION/PREGNANT	5	3.06	32.89	4.97	9.04	3.61
21	4151	FIRST INSEMINATION/PREGNANT	5					
22	4151	FIRST INSEMINATION/PREGNANT	10					
23	4151	FIRST INSEMINATION/PREGNANT	5	2.98	31.17	4.71	8.59	3.44
24	4151	FIRST INSEMINATION/PREGNANT	4	3.9	30.67	4.74	8.65	3.46
25	4151	FIRST INSEMINATION/PREGNANT	8	2.86	30.49	4.6	8.39	3.37
26	4151	FIRST INSEMINATION/PREGNANT	7	3.32	32.1	4.88	8.89	3.56
27	4151	FIRST INSEMINATION/PREGNANT	8	2.56	31.24	4.68	8.52	3.42
28	4151	FIRST INSEMINATION/PREGNANT	7					
29	4151	FIRST INSEMINATION/PREGNANT	5	2.65	24.72	3.77	6.87	2.81
30	4151	FIRST INSEMINATION/PREGNANT	7	3.3	31.87	4.85	8.83	3.53
31	4151	FIRST INSEMINATION/PREGNANT	9	4.05	31.16	4.83	8.8	3.52
32	4151	FIRST INSEMINATION/PREGNANT	5	3.03	30.9	4.68	8.53	3.42
33	4151	FIRST INSEMINATION/PREGNANT	6					
34	4151	FIRST INSEMINATION/PREGNANT	11	2.88	31	4.68	8.52	3.42
35	4151	FIRST INSEMINATION/PREGNANT	10					
36	4151	FIRST INSEMINATION/PREGNANT	10	3.34	30.92	4.72	8.6	3.45
37	4151	FIRST INSEMINATION/PREGNANT	9	3.2	30.81	4.69	8.54	3.43
38	4151	FIRST INSEMINATION/PREGNANT	8	3.63	30.99	4.76	8.68	3.48
39	4151	FIRST INSEMINATION/PREGNANT	7	3.53	30.87	4.73	8.62	3.46
40	4151	FIRST INSEMINATION/PREGNANT	8	3.29	30.53	4.66	8.49	3.41
41	4151	FIRST INSEMINATION/PREGNANT	8	4.09	31.11	4.83	8.8	3.52
42	4151	FIRST INSEMINATION/PREGNANT	9					
43	4151	FIRST INSEMINATION/PREGNANT	9	3.59	30.65	4.71	8.58	3.44
44	4151	FIRST INSEMINATION/PREGNANT	13	4.13	30.54	4.75	8.66	3.47
45	4151	FIRST INSEMINATION/PREGNANT	11	3.57	31.94	4.89	8.91	3.56
46	4151	FIRST INSEMINATION/PREGNANT	10	3.63	30.59	4.7	8.57	3.44

47	4151	FIRST INSEMINATION/PREGNANT	10	3.28	31.71	4.82	8.79	3.52
48	4151	FIRST INSEMINATION/PREGNANT	8	3.01	31.63	4.78	8.71	3.49
49	4151	FIRST INSEMINATION/PREGNANT						
50	4151	FIRST INSEMINATION/PREGNANT	13					
51	4151	FIRST INSEMINATION/PREGNANT	13	3	30.98	4.69	8.54	3.43
52	4151	FIRST INSEMINATION/PREGNANT	14	3.43	30.65	4.69	8.55	3.43
53	4151	FIRST INSEMINATION/PREGNANT	16	3.48	30.38	4.66	8.49	3.41
54	4151	FIRST INSEMINATION/PREGNANT	13	3.36	31.11	4.75	8.65	3.47
55	4151	FIRST INSEMINATION/PREGNANT	14	3.34	31.56	4.81	8.76	3.51
56	4151	FIRST INSEMINATION/PREGNANT	13					
57	4151	FIRST INSEMINATION/PREGNANT	10	3.48	29.82	4.58	8.34	3.35
58	4151	FIRST INSEMINATION/PREGNANT	10	3.28	31.54	4.8	8.74	3.5
59	4151	FIRST INSEMINATION/PREGNANT	10	3.59	31.21	4.79	8.72	3.49
60	4151	FIRST INSEMINATION/PREGNANT	12	3.26	30.23	4.61	8.4	3.38
61	4151	FIRST INSEMINATION/PREGNANT	12	3.16	30.76	4.68	8.52	3.42
62	4151	FIRST INSEMINATION/PREGNANT	11	3.13	30.49	4.63	8.44	3.39
63	4151	FIRST INSEMINATION/PREGNANT						
64	4151	FIRST INSEMINATION/PREGNANT	12	2.86	29.31	4.44	8.09	3.26
65	4151	FIRST INSEMINATION/PREGNANT	12	2.93	30.4	4.6	8.38	3.37

n = 3

Day	cow number	state	weather temperature (Celsius)	Fat %	Density%	Lactose%	SNF%	Protein%
1	3936	NON-PREGNANT	11	3.73	26.07	4.08	7.44	3.02
2	3936	NON-PREGNANT	11	4.17	29.01	4.54	8.28	3.33
3	3936	NON-PREGNANT	10	3.07	30.38	4.61	8.4	3.38
4	3936	NON-PREGNANT	11	2.55	29.71	4.46	8.13	3.27
5	3936	NON-PREGNANT	9	2.24	30.4	4.53	8.24	3.32
6	3936	NON-PREGNANT	8	3.92	28.84	4.49	8.18	3.29
7	3936	NON-PREGNANT	10					
8	3936	NON-PREGNANT	9	2.55	29.71	4.46	8.13	3.27
9	3936	NON-PREGNANT	11	2.43	29.69	4.45	8.09	3.26
10	3936	NON-PREGNANT	8	2.03	30.29	4.49	8.17	3.29
11	3936	NON-PREGNANT	7	3.41	30.07	4.61	8.39	3.37
12	3936	NON-PREGNANT	10	2.96	29.53	4.48	8.16	3.29
13	3936	FIRST INSEMINATION	10	3.19	29.35	4.48	8.17	3.29
14	3936	FIRST INSEMINATION	9					
15	3936	FIRST INSEMINATION	6					
16	3936	FIRST INSEMINATION	5	2.68	30.89	4.64	8.45	3.39
17	3936	FIRST INSEMINATION	3	2.09	30.68	4.55	8.28	3.33
18	3936	FIRST INSEMINATION	6	2.35	30.64	4.57	8.32	3.35
19	3936	FIRST INSEMINATION	4	2.5	29.77	4.47	8.13	3.28
20	3936	FIRST INSEMINATION	5	2.32	30.78	4.59	8.35	3.36
21	3936	FIRST INSEMINATION	5					
22	3936	FIRST INSEMINATION	10					
23	3936	FIRST INSEMINATION	5	2.88	29.76	4.5	8.2	3.3

24	3936	FIRST INSEMINATION	4	3.61	29.89	4.6	8.39	3.37
25	3936	FIRST INSEMINATION	8	1.96	30.63	4.53	8.24	3.32
26	3936	FIRST INSEMINATION	7	2.88	29.68	4.49	8.19	3.3
27	3936	FIRST INSEMINATION	8	3.11	30.19	4.59	8.36	3.36
28	3936	FIRST INSEMINATION	7					
29	3936	FIRST INSEMINATION	5	2.01	29.8	4.42	8.04	3.24
30	3936	FIRST INSEMINATION	7	2.39	29.72	4.45	8.09	3.26
31	3936	FIRST INSEMINATION	9	3.5	29.02	4.47	8.14	3.28
32	3936	FIRST INSEMINATION	5	4.12	28.78	4.5	8.21	3.3
33	3936	FIRST INSEMINATION	6	2.74	29.14	4.4	8.02	3.24
34	3936	FIRST INSEMINATION	11	3.49	29.87	4.59	8.36	3.36
35	3936	FIRST INSEMINATION	10					
36	3936	FIRST INSEMINATION	10	2.11	30.16	4.48	8.15	3.28
37	3936	FIRST INSEMINATION	9	2.86	28.82	4.37	7.96	3.21
38	3936	SECOND INSEMINATION	8	2.27	29.2	4.36	7.94	3.2
39	3936	SECOND INSEMINATION	7	3.84	29.34	4.55	8.3	3.34
40	3936	SECOND INSEMINATION	8	2.68	30.54	4.59	8.36	3.36
41	3936	THIRD INSEMINATION	8	4.22	30.05	4.69	8.56	3.43
42	3936	THIRD INSEMINATION	9					
43	3936	THIRD INSEMINATION	9	2.46	30.88	4.62	8.41	3.38
44	3936	THIRD INSEMINATION	13	2.79	30.38	4.58	8.35	3.35
45	3936	THIRD INSEMINATION	11	1.82	31.4	4.62	8.41	3.38
46	3936	THIRD INSEMINATION	10	4.34	30.22	4.73	8.62	3.46
47	3936	THIRD INSEMINATION	10	2.98	30.09	4.56	8.31	3.34
48	3936	THIRD INSEMINATION	8	2.99	30.18	4.58	8.34	3.35
49	3936	THIRD INSEMINATION						
50	3936	THIRD INSEMINATION	13					

51	3936	THIRD INSEMINATION	13	2.82	30.12	4.55	8.28	3.33
52	3936	THIRD INSEMINATION	14	2.33	29.89	4.46	8.13	3.27
53	3936	THIRD INSEMINATION	16	3.06	29.54	4.49	8.19	3.3
54	3936	THIRD INSEMINATION	13	2.51	30.66	4.59	8.36	3.36
55	3936	THIRD INSEMINATION	14	3.6	29.08	4.49	8.18	3.29
56	3936	THIRD INSEMINATION	13					
57	3936	THIRD INSEMINATION	10	3.54	30.35	4.66	8.49	3.41
58	3936	THIRD INSEMINATION	10	3.38	29.6	4.54	8.27	3.33
59	3936	THIRD INSEMINATION	10	4.21	30.01	4.68	8.54	3.43
60	3936	THIRD INSEMINATION	12	2.3	30.15	4.5	8.19	3.3
61	3936	THIRD INSEMINATION	12	3.16	29.47	4.49	8.19	3.3
62	3936	THIRD INSEMINATION	11	1.21	30.07	4.37	7.94	3.21
63	3936	THIRD INSEMINATION						
64	3936	THIRD INSEMINATION	12	3.48	29.77	4.57	8.33	3.35
65	3936	THIRD INSEMINATION	12	3.31	29.52	4.52	8.23	3.31

n = 4

Day	cow number	state	weather temperature (Celsius)	Fat %	Density%	Lactose%	SNF%	Protein%
1	4176	NON-PREGNANT	11	3.33	29.19	4.47	8.15	3.28
2	4176	NON-PREGNANT	11	2.9	30.69	4.64	8.45	3.39
3	4176	NON-PREGNANT	10	2.15	30.32	4.5	8.2	3.3
4	4176	FIRST INSEMINATION	11	3.15	30.74	4.67	8.51	3.42
5	4176	FIRST INSEMINATION	9	4.61	29.31	4.63	8.45	3.39
6	4176	FIRST INSEMINATION	8	3.98	31.05	4.81	8.76	3.51
7	4176	FIRST INSEMINATION	10					

8	4176	FIRST INSEMINATION	9	3.27	29.74	4.54	8.28	3.33
9	4176	FIRST INSEMINATION	11	3.55	30.84	4.73	8.62	3.45
10	4176	FIRST INSEMINATION	8	4.08	30.05	4.68	8.53	3.42
11	4176	FIRST INSEMINATION	7	3.22	29.65	4.53	8.25	3.32
12	4176	FIRST INSEMINATION	10	3.15	29.78	4.54	8.27	3.33
13	4176	FIRST INSEMINATION	10	0.93	29.77	4.3	7.81	3.16
14	4176	FIRST INSEMINATION	9					
15	4176	FIRST INSEMINATION	6	2.79	30.94	4.66	8.49	3.41
16	4176	FIRST INSEMINATION	5	3.35	30.52	4.66	8.5	3.41
17	4176	FIRST INSEMINATION	3	2.91	31.09	4.7	8.55	3.43
18	4176	FIRST INSEMINATION	6	4.19	29.61	4.62	8.44	3.39
19	4176	FIRST INSEMINATION	4	3.27	29.97	4.58	8.34	3.35
20	4176	FIRST INSEMINATION	5	6.01	30.44	4.94	9.02	3.6
21	4176	FIRST INSEMINATION	5					
22	4176	FIRST INSEMINATION	10					
23	4176	FIRST INSEMINATION	5	3.42	30.16	4.62	8.42	3.38
24	4176	FIRST INSEMINATION	4	4.46	30.37	4.76	8.69	3.48
25	4176	SECOND INSEMINATION	8					
26	4176	SECOND INSEMINATION	7	3.89	30.58	4.73	8.62	3.46
27	4176	SECOND INSEMINATION	8	3.87	30.81	4.76	8.68	3.48
28	4176	SECOND INSEMINATION	7					
29	4176	SECOND INSEMINATION	5	3.31	30.27	4.62	8.43	3.38
30	4176	SECOND INSEMINATION	7	4.3	29.88	4.67	8.53	3.42
31	4176	SECOND INSEMINATION	9	3.93	30.62	4.74	8.64	3.46
32	4176	SECOND INSEMINATION	5	3.52	29.98	4.6	8.39	3.37
33	4176	SECOND INSEMINATION	6	3.04	30.24	4.59	8.36	3.36
34	4176	SECOND INSEMINATION	11	3.24	30.24	4.61	8.4	3.37
35	4176	SECOND INSEMINATION	10					

36	4176	SECOND INSEMINATION	10	3.41	30.65	4.69	8.54	3.43
37	4176	SECOND INSEMINATION	9	3.65	30.97	4.76	8.67	3.47
38	4176	SECOND INSEMINATION	8	3.1	30.23	4.6	8.37	3.36
39	4176	SECOND INSEMINATION	7	3.47	30.06	4.61	8.4	3.38
40	4176	SECOND INSEMINATION	8	2.82	30.52	4.6	8.39	3.37
41	4176	THIRD INSEMINATION	8	3.56	31.07	4.76	8.68	3.48
42	4176	THIRD INSEMINATION	9					
43	4176	THIRD INSEMINATION	9	3.61	30.27	4.66	8.49	3.41
44	4176	THIRD INSEMINATION	13	3.66	29.93	4.61	8.41	3.38
45	4176	THIRD INSEMINATION	11	3.41	30.11	4.61	8.41	3.38
46	4176	THIRD INSEMINATION	10	3.06	30.48	4.62	8.43	3.38
47	4176	THIRD INSEMINATION	10	3.42	30.16	4.62	8.42	3.38
48	4176	FOURTH INSEMINATION	8	3.42	27.39	4.23	7.71	3.12
49	4176	FOURTH INSEMINATION						
50	4176	FOURTH INSEMINATION	13					
51	4176	FOURTH INSEMINATION	13	3.69	31.03	4.77	8.7	3.48
52	4176	FOURTH INSEMINATION	14	3.34	30.62	4.68	8.52	3.42
53	4176	FOURTH INSEMINATION	16	3.3	30.37	4.64	8.46	3.39
54	4176	FOURTH INSEMINATION	13	3.91	30.38	4.7	8.58	3.44
55	4176	FOURTH INSEMINATION	14	3.63	30.08	4.63	8.44	3.39
56	4176	FOURTH INSEMINATION	13					
57	4176	FOURTH INSEMINATION	10	3.08	28.97	4.41	8.04	3.24
58	4176	FOURTH INSEMINATION	10	3.47	30.5	4.67	8.52	3.42
59	4176	FOURTH INSEMINATION	10	3.59	30.67	4.71	8.58	3.44
60	4176	FOURTH INSEMINATION	12	3.38	30.03	4.6	8.38	3.37
61	4176	FOURTH INSEMINATION	12	3.24	30.21	4.61	8.4	3.37
62	4176	FOURTH INSEMINATION	11					
63	4176	FOURTH INSEMINATION						

64	4176	FOURTH INSEMINATION	12	3.74	30.53	4.71	8.58	3.44
65	4176	FOURTH INSEMINATION	12	3.79	29.44	4.56	8.31	3.34

n = 5

Day	cow number	state	weather temperature (Celsius)	FAT%	Density%	Lactose%	SNF%	Protein%
1	4049	NON-PREGNANT	11	3.51	28.97	4.46	8.13	3.28
2	4049	NON-PREGNANT	11	2.85	29.83	4.51	8.22	3.31
3	4049	NON-PREGNANT	10	3.12	29.64	4.51	8.23	3.31
4	4049	NON-PREGNANT	11	4.15	28.63	4.48	8.18	3.29
5	4049	NON-PREGNANT	9	3.86	28.99	4.5	8.21	3.3
6	4049	NON-PREGNANT	8	3.82	28.43	4.42	8.06	3.25
7	4049	NON-PREGNANT	10					
8	4049	NON-PREGNANT	9	1.65	29.79	4.38	7.96	3.21
9	4049	NON-PREGNANT	11	2.62	28.79	4.34	7.9	3.19
10	4049	NON-PREGNANT	8	2.83	29.81	4.51	8.21	3.3
11	4049	NON-PREGNANT	7	3.21	28.97	4.43	8.07	3.25
12	4049	NON-PREGNANT	10	3.68	28.69	4.44	8.1	3.26
13	4049	FIRST INSEMINATION	10	3.96	28.81	4.49	8.18	3.29
14	4049	FIRST INSEMINATION	9					
15	4049	FIRST INSEMINATION	6	3.06	29.12	4.43	8.08	3.26
16	4049	FIRST INSEMINATION	5	3.61	28.74	4.44	8.1	3.26
17	4049	FIRST INSEMINATION	3	3.46	29.85	4.58	8.35	3.35
18	4049	FIRST INSEMINATION	6	2.97	29.22	4.44	8.09	3.26
19	4049	FIRST INSEMINATION	4	3.01	29.64	4.5	8.2	3.3

20	4049	FIRST INSEMINATION	5	3.37	29.77	4.56	8.31	3.34
21	4049	FIRST INSEMINATION	5					
22	4049	FIRST INSEMINATION	10					
23	4049	FIRST INSEMINATION	5	3.79	29.84	4.61	8.41	3.38
24	4049	FIRST INSEMINATION	4	3.84	29.72	4.6	8.39	3.37
25	4049	FIRST INSEMINATION	8	3.7	30.18	4.65	8.48	3.4
26	4049	FIRST INSEMINATION	7	2.56	29.95	4.5	8.19	1.15
27	4049	FIRST INSEMINATION	8	2.2	30.17	4.49	8.17	3.29
28	4049	FIRST INSEMINATION	7					
29	4049	FIRST INSEMINATION	5	3.47	28.63	4.41	8.04	3.24
30	4049	FIRST INSEMINATION	7	4.17	28.49	4.46	8.15	3.28
31	4049	FIRST INSEMINATION	9	4.17	29.45	4.6	8.39	3.37
32	4049	FIRST INSEMINATION	5	4.64	28.34	4.49	8.2	3.3
33	4049	FIRST INSEMINATION	6	4.09	29.77	4.64	8.46	3.39
34	4049	FIRST INSEMINATION	11	4.19	29.96	4.67	8.58	3.42
35	4049	FIRST INSEMINATION	10					
36	4049	FIRST INSEMINATION	10	3.07	30.36	4.61	8.4	3.37
37	4049	FIRST INSEMINATION	9	3.15	29.57	4.51	8.21	3.31
38	4049	SECOND INSEMINATION	8	2.76	29.59	4.47	8.14	3.28
39	4049	SECOND INSEMINATION	7	4.25	29.23	4.58	8.35	3.36
40	4049	SECOND INSEMINATION	8	3.89	29.5	4.58	8.35	3.35
41	4049	SECOND INSEMINATION	8	4.51	30.1	4.73	8.63	3.46
42	4049	SECOND INSEMINATION	9					
43	4049	SECOND INSEMINATION	9	3.02	30.97	4.69	8.55	3.43
44	4049	SECOND INSEMINATION	13	3.02	30.46	4.62	8.41	3.38
45	4049	SECOND INSEMINATION	11	2.99	30.35	4.6	8.38	3.37
46	4049	SECOND INSEMINATION	10	2.85	30.35	4.58	8.35	3.36

47	4049	SECOND INSEMINATION	10	2.89	29.45	4.46	8.13	3.27
48	4049	SECOND INSEMINATION	8	3.16	30.27	4.61	8.39	3.37
49	4049	SECOND INSEMINATION						
50	4049	SECOND INSEMINATION	13					
51	4049	SECOND INSEMINATION	13	3.22	29.32	4.48	8.16	3.29
52	4049	SECOND INSEMINATION	14	3.92	29.54	4.59	8.36	3.36
53	4049	SECOND INSEMINATION	16	3.34	29.28	4.49	8.18	3.29
54	4049	SECOND INSEMINATION	13	3.11	30.12	4.58	8.34	3.35
55	4049	SECOND INSEMINATION	14	4.01	29.08	4.53	8.26	3.32
56	4049	SECOND INSEMINATION	13					
57	4049	SECOND INSEMINATION	10	2.8	30.07	4.54	8.27	3.33
58	4049	SECOND INSEMINATION	10	3.73	30.58	4.71	8.59	3.44
59	4049	SECOND INSEMINATION	10	4.26	30.61	4.77	8.71	3.49
60	4049	SECOND INSEMINATION	12	3.47	28.6	4.40	8.03	3.24
61	4049	SECOND INSEMINATION	12	5.5	28.74	4.64	8..48	3.4
62	4049	SECOND INSEMINATION	11	3.17	29.19	4.46	8.12	3.27
63	4049	SECOND INSEMINATION						
64	4049	SECOND INSEMINATION	12	3.14	29.29	4.47	8.14	3.28
65	4049	SECOND INSEMINATION	12	2.79	29.09	4.4	8.02	3.23

n = 6 / Measured during 34 days, extracted from group due to illness and death

Day	cow number	state	weather temperature (Celsius)	FAT%	Density%	Lactose%	SNF%	Protein%
1	3916	NON-PREGNANT	11	2.01	29.75	4.41	8.03	3.24
2	3916	NON-PREGNANT	11	3.94	27.82	4.35	7.93	3.2
3	3916	NON-PREGNANT	10	3.59	27.25	4.23	7.71	3.12
4	3916	NON-PREGNANT	11	4.35	26.75	4.24	7.74	3.13
5	3916	NON-PREGNANT	9	4.39	26.53	4.21	7.69	3.11
6	3916	NON-PREGNANT	8	3.94	26.72	4.19	7.65	3.1
7	3916	NON-PREGNANT	10					
8	3916	NON-PREGNANT	9	3.56	27.53	4.26	7.78	3.15
9	3916	NON-PREGNANT	11	3.19	27.9	4.28	7.79	3.15
10	3916	NON-PREGNANT	8	3.76	27.88	4.33	7.9	3.19
11	3916	NON-PREGNANT	7	4.2	27.24	4.29	7.83	3.17
12	3916	NON-PREGNANT	10					
13	3916	FIRST INSEMINATION	10	4.28	27.6	4.35	7.94	3.21
14	3916	FIRST INSEMINATION	9					
15	3916	FIRST INSEMINATION	6	3.71	27.58	4.29	7.82	3.16
16	3916	FIRST INSEMINATION	5	6.88	26.43	4.47	8.18	3.29
17	3916	FIRST INSEMINATION	3	3.73	28.16	4.37	7.97	3.22
18	3916	FIRST INSEMINATION	6	3.96	27.66	4.32	7.89	3.19
19	3916	FIRST INSEMINATION	4	4.19	25.86	4.09	7.48	3.03
20	3916	FIRST INSEMINATION	5	4.95	27.43	4.4	8.03	3.24
21	3916	FIRST INSEMINATION	5					
22	3916	FIRST INSEMINATION	10					
23	3916	FIRST INSEMINATION	5	4.81	26.63	4.27	7.8	3.15

24	3916	FIRST INSEMINATION	4	4.08	27.1	4.26	7.77	3.14
25	3916	FIRST INSEMINATION	8	1.02	27.22	3.95	7.17	2.93
26	3916	FIRST INSEMINATION	7	5.69	26.3	4.32	7.9	3.19
27	3916	FIRST INSEMINATION	8	4.77	26.26	4.21	7.7	3.12
28	3916	FIRST INSEMINATION	7					
29	3916	SICK	5	3.95	20.79	3.35	6.13	2.54
30	3916	FIRST INSEMINATION	7	4.32	27.17	4.3	7.84	3.17
31	3916	FIRST INSEMINATION	9	4.63	25.93	4.15	7.59	3.07
32	3916	FIRST INSEMINATION	5	5.55	26.13	4.28	7.83	3.16
33	3916	FIRST INSEMINATION	6	4.63	25.93	4.15	7.59	3.07
34	3916	FIRST INSEMINATION	11	5.55	26.13	4.28	7.83	3.16

n = 7

Day	cow number	state	weather temperature (Celsius)	Fat%	Density%	Lactose%	SNF%	Protein%
1	4143	NON-PREGNANT	11	3.87	29.83	4.62	8.43	3.38
2	4143	NON-PREGNANT	11	2.89	30.76	4.65	8.46	3.4
3	4143	NON-PREGNANT	10	2.21	30.66	4.56	8.3	3.34
4	4143	NON-PREGNANT	11	3.67	29.27	4.58	8.35	3.36
5	4143	NON-PREGNANT	9	4.51	29.35	4.62	8.44	3.39
6	4143	NON-PREGNANT	8	2.53	31.41	4.7	8.55	3.43
7	4143	NON-PREGNANT	10					
8	4143	NON-PREGNANT	9	3.53	29.86	4.59	8.37	3.36
9	4143	NON-PREGNANT	11	4.11	30.89	4.8	8.75	3.5
10	4143	NON-PREGNANT	8	3.84	30.3	4.68	8.54	3.43

11	4143	NON-PREGNANT	7	3.8	30.54	4.71	8.6	3.45
12	4143	NON-PREGNANT	10	4.01	29.97	4.66	8.49	3.41
13	4143	FIRST INSEMINATION	10					
14	4143	FIRST INSEMINATION	9					
15	4143	FIRST INSEMINATION	6	3.86	29.03	4.51	8.22	3.31
16	4143	FIRST INSEMINATION	5	4.27	29.41	4.6	8.4	3.37
17	4143	FIRST INSEMINATION	3	3.18	30.95	4.71	8.57	3.44
18	4143	FIRST INSEMINATION	6	3.3	30.18	4.61	8.4	3.37
19	4143	FIRST INSEMINATION	4	3.5	31.46	4.81	8.77	3.51
20	4143	FIRST INSEMINATION	5	3.73	31.16	4.79	8.74	3.5
21	4143	FIRST INSEMINATION	5					
22	4143	FIRST INSEMINATION	10					
23	4143	FIRST INSEMINATION	5	3.55	30.63	4.7	8.57	3.44
24	4143	FIRST INSEMINATION	4	3.65	29.7	4.58	8.35	3.36
25	4143	FIRST INSEMINATION	8	3.12	29.72	4.52	8.24	3.32
26	4143	FIRST INSEMINATION	7	3.89	29.69	4.6	8.39	3.37
27	4143	FIRST INSEMINATION	8	3.57	29.69	4.57	8.33	3.35
28	4143	FIRST INSEMINATION	7					
29	4143	FIRST INSEMINATION	5	4.14	30.07	4.68	8.54	3.43
30	4143	FIRST INSEMINATION	7	3.7	30	4.63	8.43	3.39
31	4143	FIRST INSEMINATION	9	3.89	30.93	4.78	8.71	3.49
32	4143	FIRST INSEMINATION	5	3.85	29.5	4.57	8.34	3.35
33	4143	FIRST INSEMINATION	6	3.75	29.07	4.5	8.21	3.3
34	4143	FIRST INSEMINATION	11	3.47	29.75	4.57	8.32	3.35
35	4143	FIRST INSEMINATION	10					
36	4143	FIRST INSEMINATION	10					
37	4143	FIRST INSEMINATION	9	3.56	30.35	4.66	8.5	3.41

38	4143	SECOND INSEMINATION	8	3.83	30.45	4.7	8.58	3.44
39	4143	SECOND INSEMINATION	7	3.71	30.44	4.69	8.55	3.43
40	4143	SECOND INSEMINATION	8	3.42	30.35	4.65	8.47	3.4
41	4143	SECOND INSEMINATION	8	4.23	30.38	4.74	8.64	3.46
42	4143	SECOND INSEMINATION	9					
43	4143	SECOND INSEMINATION	9	3.8	30.5	4.71	8.58	3.44
44	4143	SECOND INSEMINATION	13	3.69	29.91	4.61	8.41	3.38
45	4143	SECOND INSEMINATION	11	3.87	30.18	4.67	8.52	3.42
46	4143	SECOND INSEMINATION	10	3.63	29.89	4.6	8.39	3.37
47	4143	SECOND INSEMINATION	10	3.7	29.62	4.57	8.34	3.35
48	4143	SECOND INSEMINATION	8	3.74	30.53	4.71	8.58	3.44
49	4143	SECOND INSEMINATION						
50	4143	SECOND INSEMINATION	13					
51	4143	SECOND INSEMINATION	13	3.95	30	4.65	8.49	3.41
52	4143	SECOND INSEMINATION	14	3.35	26.55	4.75	8.71	3.48
53	4143	SECOND INSEMINATION	16	3.36	29.47	4.51	8.23	3.31
54	4143	SECOND INSEMINATION	13	3.74	30.69	4.73	8.62	3.46
55	4143	SECOND INSEMINATION	14	4.13	29.79	4.64	8.47	3.4
56	4143	SECOND INSEMINATION	13					
57	4143	SECOND INSEMINATION	10	3.63	29.52	4.55	8.3	3.34
58	4143	SECOND INSEMINATION	10	3.91	30.6	4.73	8.63	3.46
59	4143	SECOND INSEMINATION	10	4.10E+00	30.12	4.69E+00	8.55	3.43
60	4143	SECOND INSEMINATION	12	3.97E+00	29.84	4.63E+00	8.45	3.39
61	4143	SECOND INSEMINATION	12	3.51E+00	29.16	4.49	8.18	3.29
62	4143	SECOND INSEMINATION	11	3.86	29.57	4.58	8.36	3.36
63	4143	SECOND INSEMINATION						
64	4143	SECOND INSEMINATION	12	5.29	29.25	4.69	8.57	3.43

65	4143	SECOND INSEMINATION	12	3.89	30.04	4.65	8.48	3.4

n = 8

Day	cow number	state	weather temperature (Celsius)	Fat%	Density%	Lactose%	SNF%	Protein%
1	4058	NON-PREGNANT	11	2.89	30.6	4.62	8.42	3.38
2	4058	NON-PREGNANT	11	2.7	30.67	4.61	8.4	3.38
3	4058	NON-PREGNANT	10	5.11	29.68	4.73	8.64	3.46
4	4058	NON-PREGNANT	11	4.36	29.26	4.59	8.38	3.37
5	4058	NON-PREGNANT	9	4.03	29.99	4.66	8.5	3.41
6	4058	NON-PREGNANT	8	3.8	30.52	4.71	8.59	3.44
7	4058	NON-PREGNANT	10					
8	4058	NON-PREGNANT	9	4.32	30.39	4.75	8.66	3.47
9	4058	NON-PREGNANT	11	4.04	30.32	4.71	8.59	3.44
10	4058	NON-PREGNANT	8	3.96	30.66	4.75	8.66	3.47
11	4058	NON-PREGNANT	7	3.85	30.25	4.68	8.53	3.42
12	4058	NON-PREGNANT	10	3.52	30.21	4.64	8.45	3.39
13	4058	NON-PREGNANT	10	3.91	30.01	4.65	8.48	3.4
14	4058	NON-PREGNANT	9					
15	4058	NON-PREGNANT	6	3.68	30.12	4.64	8.46	3.4
16	4058	NON-PREGNANT	5	4.28	30.02	4.69	8.56	3.43
17	4058	NON-PREGNANT	3	4.18	30.83	4.8	8.75	3.5
18	4058	NON-PREGNANT	6	3.93	30.36	4.7	8.58	3.44
19	4058	NON-PREGNANT	4	3.93	30.22	4.68	8.54	3.42
20	4058	NON-PREGNANT	5	4.84	30.33	4.8	8.75	3.5

21	4058	NON-PREGNANT	5					
22	4058	NON-PREGNANT	10					
23	4058	NON-PREGNANT	5	4.61	30.69	4.82	8.8	3.52
24	4058	NON-PREGNANT	4	3.93	30.22	4.68	8.54	3.42
25	4058	NON-PREGNANT	8	3.92	30.27	4.69	8.55	3.43
26	4058	NON-PREGNANT	7	3.96	30.14	4.67	8.53	3.42
27	4058	NON-PREGNANT	8	3.39	31.08	4.75	8.65	3.46
28	4058	NON-PREGNANT	7					
29	4058	NON-PREGNANT	5	4.96	30.87	4.89	8.92	3.56
30	4058	FIRST INSEMINATION	7	4.71	30.44	4.8	8.76	3.5
31	4058	FIRST INSEMINATION	9	4.65	30.8	4.84	8.84	3.53
32	4058	FIRST INSEMINATION	5	4.41	29.79	4.67	8.53	3.42
33	4058	FIRST INSEMINATION	6	3.86	30.89	4.77	8.7	3.48
34	4058	FIRST INSEMINATION	11	4.27	31.05	4.84	8.82	3.53
35	4058	FIRST INSEMINATION	10					
36	4058	FIRST INSEMINATION	10	3.84	30.14	4.66	8.5	3.41
37	4058	FIRST INSEMINATION	9	3.64	30.33	4.67	8.51	3.41
38	4058	FIRST INSEMINATION	8	4.09	30.4	4.73	8.62	3.45
39	4058	FIRST INSEMINATION	7	4.19	30.01	4.68	8.54	3.42
40	4058	FIRST INSEMINATION	8	4.47	30.29	4.75	8.67	3.47
41	4058	FIRST INSEMINATION	8	4.9	30.34	4.8	8.77	3.51
42	4058	FIRST INSEMINATION	9					
43	4058	FIRST INSEMINATION	9	4.18	31.42	4.88	8.9	3.56
44	4058	FIRST INSEMINATION	13	4.02	31.45	4.87	8.87	3.55
45	4058	FIRST INSEMINATION	11	3.98	30.47	4.72	8.61	3.45
46	4058	FIRST INSEMINATION	10	4.08	30.08	4.68	8.53	3.42
47	4058	FIRST INSEMINATION	10	3.82	30.73	4.74	8.65	3.46

48	4058	FIRST INSEMINATION	8	3.99	30.79	4.77	8.7	3.48
49	4058	FIRST INSEMINATION						
50	4058	FIRST INSEMINATION	13					
51	4058	FIRST INSEMINATION	13	4.16	31.21	4.85	8.84	3.54
52	4058	SECOND INSEMINATION	14	3.47	30.81	4.72	8.6	3.45
53	4058	SECOND INSEMINATION	16	4.19	30.5	4.75	8.67	3.47
54	4058	SECOND INSEMINATION	13	4.48	30.97	4.85	8.85	3.54
55	4058	SECOND INSEMINATION	14	4.89	30.06	4.76	8.7	3.48
56	4058	SECOND INSEMINATION	13					
57	4058	SECOND INSEMINATION	10	4.21	30.17	4.71	8.58	3.44
58	4058	SECOND INSEMINATION	10	4.06	30.45	4.73	8.63	3.46
59	4058	SECOND INSEMINATION	10	6.49	30.15	4.95	9.05	3.61
60	4058	SECOND INSEMINATION	12	4.1	30.12	4.69	8.55	3.43
61	4058	SECOND INSEMINATION	12	3.71	30.02	4.63	8.44	3.39
62	4058	SECOND INSEMINATION	11	3.83	30.82	4.76	8.67	3.47
63	4058	SECOND INSEMINATION						
64	4058	SECOND INSEMINATION	12	4.48	31	4.85	8.85	3.54
65	4058	SECOND INSEMINATION	12	4.31	30.53	4.77	8.7	3.48

n = 9

Day	cow number	state	weather temperature (Celsius)	FAT%	Density%	Lactose%	SNF%	Protein%
1	4169	NON-PREGNANT	11	1.97	29.36	4.35	7.92	3.2
2	4169	NON-PREGNANT	11	3.51	29.37	4.52	8.24	3.31
3	4169	NON-PREGNANT	10	3.08	29.51	4.49	8.18	3.29

4	4169	NON-PREGNANT	11	3.16	27.25	4.18	7.69	3.09
5	4169	NON-PREGNANT	9	3.16	27.25	4.18	7.69	3.09
6	4169	NON-PREGNANT	8	2.72	28.8	4.35	7.93	3.2
7	4169	NON-PREGNANT	10					
8	4169	NON-PREGNANT	9	3.93	28.29	4.41	8.05	3.24
9	4169	NON-PREGNANT	11	2.64	29.04	4.38	7.97	3.22
10	4169	NON-PREGNANT	8	4.02	29.01	4.52	8.25	3.32
11	4169	NON-PREGNANT	7					
12	4169	NON-PREGNANT	10	3.03	28.84	4.39	8	3.23
13	4169	NON-PREGNANT	10	3.41	29.18	4.45	8.11	3.27
14	4169	NON-PREGNANT	9					
15	4169	NON-PREGNANT	6	2.8	29.13	4.41	8.03	3.24
16	4169	NON-PREGNANT	5	2.55	29.34	4.41	8.03	3.24
17	4169	NON-PREGNANT	3	2.73	29.99	4.52	8.23	3.31
18	4169	NON-PREGNANT	6	1.85	29.7	4.4	8	3.23
19	4169	NON-PREGNANT	4	2.19	29.02	4.4	8.02	3.24
20	4169	NON-PREGNANT	5	3.33	29.78	4.55	8.3	3.34
21	4169	NON-PREGNANT	5					
22	4169	NON-PREGNANT	10					
23	4169	NON-PREGNANT	5	3.01	29.76	4.52	8.23	3.31
24	4169	NON-PREGNANT	4	3.54	29.79	4.58	8.35	3.36
25	4169	NON-PREGNANT	8	2.93	29.61	4.49	8.18	3.29
26	4169	NON-PREGNANT	7	3.59	29.1	4.49	8.18	3.29
27	4169	NON-PREGNANT	8	2.2	29.25	4.36	7.94	3.21
28	4169	NON-PREGNANT	7					
29	4169	NON-PREGNANT	5	3.08	28.5	4.35	7.93	3.2
30	4169	NON-PREGNANT	7	2.56	28.47	4.29	7.81	3.16

31	4169	NON-PREGNANT	9	2.68	28.38	4.29	7.81	3.16
32	4169	NON-PREGNANT	5	3.22	28.53	4.37	7.96	3.21
33	4169	NON-PREGNANT	6	2.71	29.29	4.42	8.06	3.25
34	4169	NON-PREGNANT	11	2.87	30.53	4.61	8.4	3.38
35	4169	NON-PREGNANT	10					
36	4169	NON-PREGNANT	10	3.47	27.59	4.26	7.77	3.14
37	4169	NON-PREGNANT	9	2.6	28.84	4.34	7.91	3.2
38	4169	NON-PREGNANT	8	2.44	28.82	4.32	7.87	3.18
39	4169	NON-PREGNANT	7	3.45	28.63	4.41	8.03	3.24
40	4169	NON-PREGNANT	8	3.2	28.72	4.39	8	3.23
41	4169	FIRST INSEMINATION	8	2.55	32.48	4.85	8.83	3.53
42	4169	FIRST INSEMINATION	9					
43	4169	FIRST INSEMINATION	9	2.57	29.85	4.48	8.17	3.29
44	4169	FIRST INSEMINATION	13	2.45	29.68	4.45	8.1	3.26
45	4169	FIRST INSEMINATION	11	2.61	30.06	4.52	8.23	3.31
46	4169	FIRST INSEMINATION	10	4.62	27.92	4.43	8.09	3.26
47	4169	FIRST INSEMINATION	10	2.54	30	4.5	8.2	3.3
48	4169	FIRST INSEMINATION	8	3.2	30.5	4.58	8.35	3.35
49	4169	FIRST INSEMINATION						
50	4169	FIRST INSEMINATION	13					
51	4169	FIRST INSEMINATION	13	2.54	29.22	4.39	8	3.23
52	4169	FIRST INSEMINATION	14	2.24	30.05	4.48	8.15	3.28
53	4169	FIRST INSEMINATION	16	2.97	28.68	4.36	7.95	3.21
54	4169	FIRST INSEMINATION	13	2.28	29.8	4.45	8.09	3.26
55	4169	FIRST INSEMINATION	14	2.85	29.27	4.43	8.07	3.25
56	4169	FIRST INSEMINATION	13					
57	4169	FIRST INSEMINATION	10	3.15	28.47	4.35	7.93	3.2

58	4169	FIRST INSEMINATION	10	2.85	29.27	4.43	8.07	3.25	
59	4169	FIRST INSEMINATION	10	3.06	30.29	4.6	8.38	3.37	
60	4169	FIRST INSEMINATION	12	2.35	29.16	4.36	7.94	3.21	
61	4169	FIRST INSEMINATION	12	3.02	29.33	4.46	8.13	3.27	
62	4169	FIRST INSEMINATION	11	2.27	29.9	4.46	8.12	3.27	
63	4169	FIRST INSEMINATION							
64	4169	FIRST INSEMINATION	12	2.76	29.38	4.44	8.08	3.26	
65	4169	FIRST INSEMINATION	12	2.83	29.46	4.46	8.12	3.27	

n = 10

Day	cow number	state	weather temperature (Celsius)	FAT%	Density%	Lactose%	SNF%	Protein%
1	4282	NON-PREGNANT	11	2.83	28.31	4.29	7.82	3.16
2	4282	NON-PREGNANT	11	2.78	28.74	4.35	7.92	3.2
3	4282	NON-PREGNANT	10	4.02	29.31	4.56	8.33	3.35
4	4282	NON-PREGNANT	11	2.73	29.5	4.45	8.11	3.27
5	4282	NON-PREGNANT	9	4.44	29.45	4.63	8.45	3.39
6	4282	NON-PREGNANT	8	3.01	28.68	4.37	7.96	3.21
7	4282	NON-PREGNANT	10					
8	4282	NON-PREGNANT	9	2.41	28.21	4.24	7.71	3.12
9	4282	NON-PREGNANT	11	2.73	29.07	4.39	8	3.23
10	4282	NON-PREGNANT	8	3.39	28.57	4.39	7.98	3.22
11	4282	NON-PREGNANT	7	2.64	29.02	4.37	7.97	3.22
12	4282	NON-PREGNANT	10	3.85	28.33	4.41	8.04	3.24
13	4282	NON-PREGNANT	10					

14	4282	NON-PREGNANT	9					
15	4282	NON-PREGNANT	6	4.38	27.94	4.41	8.05	3.24
16	4282	NON-PREGNANT	5	3.72	28.61	4.43	8.08	3.26
17	4282	FIRST INSEMINATION	3	1.72	29.36	4.32	7.86	3.18
18	4282	FIRST INSEMINATION	6					
19	4282	FIRST INSEMINATION	4	4.49	28.84	4.55	8.3	3.34
20	4282	FIRST INSEMINATION	5	3.51	29.56	4.54	8.28	3.33
21	4282	FIRST INSEMINATION	5					
22	4282	FIRST INSEMINATION	10					
23	4282	FIRST INSEMINATION	5	2.69	29.25	4.41	8.04	3.24
24	4282	FIRST INSEMINATION	4	3.11	30.37	4.62	8.41	3.38
25	4282	FIRST INSEMINATION	8	2.76	29.4	4.44	8.09	3.26
26	4282	FIRST INSEMINATION	7	2.77	28.74	4.35	7.92	3.2
27	4282	FIRST INSEMINATION	8	2.69	28.75	4.34	7.91	3.19
28	4282	FIRST INSEMINATION	7					
29	4282	FIRST INSEMINATION	5	3.08	29.89	4.54	8.28	3.33
30	4282	FIRST INSEMINATION	7	3.49	28.41	4.38	7.99	3.22
31	4282	FIRST INSEMINATION	9	3.52	30.33	4.65	8.48	3.4
32	4282	FIRST INSEMINATION	5	3.32	28.53	4.38	7.98	3.22
33	4282	FIRST INSEMINATION	6	3.29	28.84	4.42	8.05	3.25
34	4282	FIRST INSEMINATION	11	2.96	30.26	4.58	8.35	3.36
35	4282	FIRST INSEMINATION	10					
36	4282	FIRST INSEMINATION	10	3.1	29.51	4.49	8.19	3.3
37	4282	FIRST INSEMINATION	9	3.45	29.19	4.49	8.18	3.29
38	4282	FIRST INSEMINATION	8	3.43	30.11	4.61	8.41	3.38
39	4282	SECOND INSEMINATION	7	4.8	28.62	4.55	8.31	3.34
40	4282	SECOND INSEMINATION	8	3.32	28.93	4.43	8.08	3.26

41	4282	SECOND INSEMINATION	8	3.43	29.57	4.54	8.27	3.33
42	4282	SECOND INSEMINATION	9					
43	4282	SECOND INSEMINATION	9	3.18	28.93	4.42	8.06	3.25
44	4282	SECOND INSEMINATION	13	3.15	29.43	4.49	8.18	3.29
45	4282	SECOND INSEMINATION	11	3.11	29.36	4.47	8.15	3.28
46	4282	SECOND INSEMINATION	10	3.11	28.69	4.38	7.98	3.22
47	4282	SECOND INSEMINATION	10	2.89	29.82	4.51	8.22	3.31
48	4282	SECOND INSEMINATION	8	2.83	28.73	4.35	7.93	3.2
49	4282	SECOND INSEMINATION						
50	4282	SECOND INSEMINATION	13					
51	4282	SECOND INSEMINATION	13	2.87	29.05	4.4	8.02	3.24
52	4282	SECOND INSEMINATION	14	3.02	29.15	4.43	8.08	3.26
53	4282	SECOND INSEMINATION	16	3.02	28.96	4.41	8.03	3.24
54	4282	SECOND INSEMINATION	13	3.43	29.17	4.48	8.17	3.29
55	4282	SECOND INSEMINATION	14	3.29	28.98	4.44	8.09	3.26
56	4282	SECOND INSEMINATION	13					
57	4282	SECOND INSEMINATION	10	3.45	28.16	4.34	7.91	3.2
58	4282	SECOND INSEMINATION	10	2.49	30.08	4.51	8.21	3.3
59	4282	SECOND INSEMINATION	10	3.41	29.15	4.48	8.16	3.29
60	4282	SECOND INSEMINATION	12	4.09	28.1	4.4	8.03	3.24
61	4282	SECOND INSEMINATION	12	0.65	29.39	4.21	7.65	3.1
62	4282	SECOND INSEMINATION	11	3.53	29.11	4.48	8.17	3.29
63	4282	SECOND INSEMINATION						
64	4282	SECOND INSEMINATION	12	3.4	29.48	4.52	8.24	3.32
65	4282	SECOND INSEMINATION	12	3.36	30.24	4.62	8.43	3.38

Table S2B: Microwave Dielectric fitting parameters (Cole-Cole: dielectric strength- Δε, relaxation time τ(s), broadening parameter-α, and conductivity-σ(Siemens/m) measured weekly for 10 different cows at different reproductive states (Non-pregnant-NP, during inseminations and during confirmed pregnancy). The not measured days correspond mostly to Saturdays ,an official holiday in Israel.

n = 1

Day	cow number	state	Δε	τ (s)	α	σ (S/m)
1	4165	NON-PREGNANT	61.02	8.12E-12	0.95	0.47
2	4165	NON-PREGNANT	61.46	8.19E-12	0.97	0.52
3	4165	NON-PREGNANT	63.23	8.27E-12	0.96	0.52
4	4165	NON-PREGNANT	60.48	8.7E-12	0.98	0.47
5	4165	NON-PREGNANT	60.40	8.25E-12	0.98	0.45
6	4165	FIRST INSEMINATION/PREGNANT	61.23	8.54E-12	0.96	0.45
7	4165	FIRST INSEMINATION/PREGNANT				
8	4165	FIRST INSEMINATION/PREGNANT	60.67	7.94E-12	0.97	0.45
9	4165	FIRST INSEMINATION/PREGNANT				
10	4165	FIRST INSEMINATION/PREGNANT	58.89	7.21E-12	0.99	0.35
11	4165	FIRST INSEMINATION/PREGNANT	60.51	7.82E-12	0.98	0.43
12	4165	FIRST INSEMINATION/PREGNANT	60.60	8.07E-12	0.98	0.44
13	4165	FIRST INSEMINATION/PREGNANT	62.00	8.39E-12	0.97	0.46
14	4165	FIRST INSEMINATION/PREGNANT				
15	4165	FIRST INSEMINATION/PREGNANT	60.57	8.12E-12	0.98	0.43
16	4165	FIRST INSEMINATION/PREGNANT	61.41	8.82E-12	0.97	0.45
17	4165	FIRST INSEMINATION/PREGNANT	57.96	7.2E-12	1.00	0.35
18	4165	FIRST INSEMINATION/PREGNANT	60.03	7.3E-12	0.99	0.41
19	4165	FIRST INSEMINATION/PREGNANT	59.36	7.78E-12	0.98	0.42
20	4165	FIRST INSEMINATION/PREGNANT	60.04	8.56E-12	0.98	0.44

21	4165	FIRST INSEMINATION/PREGNANT				
22	4165	FIRST INSEMINATION/PREGNANT				
23	4165	FIRST INSEMINATION/PREGNANT	60.45	8.8E-12	0.98	0.47
24	4165	FIRST INSEMINATION/PREGNANT	59.79	8.93E-12	0.98	0.47
25	4165	FIRST INSEMINATION/PREGNANT	59.77	9.09E-12	0.98	0.45
26	4165	FIRST INSEMINATION/PREGNANT	59.25	8.97E-12	0.98	0.45
27	4165	FIRST INSEMINATION/PREGNANT	60.26	8.81E-12	0.98	0.44
28	4165	FIRST INSEMINATION/PREGNANT				
29	4165	FIRST INSEMINATION/PREGNANT	58.95	7.34E-12	0.98	0.39
30	4165	FIRST INSEMINATION/PREGNANT	60.26	8.01E-12	0.97	0.38
31	4165	FIRST INSEMINATION/PREGNANT	59.06	8.18E-12	0.98	0.43
32	4165	FIRST INSEMINATION/PREGNANT	58.80	7.98E-12	0.98	0.42
33	4165	FIRST INSEMINATION/PREGNANT	59.87	7.87E-12	0.98	0.43
34	4165	FIRST INSEMINATION/PREGNANT	60.20	7.91E-12	0.98	0.42
35	4165	FIRST INSEMINATION/PREGNANT				
36	4165	FIRST INSEMINATION/PREGNANT	63.03	9.21E-12	0.98	0.42
37	4165	FIRST INSEMINATION/PREGNANT	60.68	9.11E-12	0.97	0.47
38	4165	FIRST INSEMINATION/PREGNANT	60.03	9.21E-12	0.97	0.46
39	4165	FIRST INSEMINATION/PREGNANT	60.94	9.27E-12	0.97	0.46
40	4165	FIRST INSEMINATION/PREGNANT	61.55	9.11E-12	0.97	0.47
41	4165	FIRST INSEMINATION/PREGNANT	59.62	9.06E-12	0.97	0.46
42	4165	FIRST INSEMINATION/PREGNANT				
43	4165	FIRST INSEMINATION/PREGNANT	60.54	9.5E-12	0.97	0.46
44	4165	FIRST INSEMINATION/PREGNANT				
45	4165	FIRST INSEMINATION/PREGNANT	60.47	8.94E-12	0.97	0.48
46	4165	FIRST INSEMINATION/PREGNANT	60.61	9.01E-12	0.97	0.48

47	4165	FIRST INSEMINATION/PREGNANT	60.99	8.99E-12	0.96	0.48
48	4165	FIRST INSEMINATION/PREGNANT	61.53	8.31E-12	0.97	0.44
49	4165	FIRST INSEMINATION/PREGNANT				
50	4165	FIRST INSEMINATION/PREGNANT				
51	4165	FIRST INSEMINATION/PREGNANT	59.41	8.62E-12	0.98	0.46
52	4165	FIRST INSEMINATION/PREGNANT	58.38	8.76E-12	0.98	0.46
53	4165	FIRST INSEMINATION/PREGNANT	59.94	8.73E-12	0.97	0.47
54	4165	FIRST INSEMINATION/PREGNANT	59.97	8.72E-12	0.97	0.45
55	4165	FIRST INSEMINATION/PREGNANT	60.15	8.93E-12	0.97	0.45
56	4165	FIRST INSEMINATION/PREGNANT				
57	4165	FIRST INSEMINATION/PREGNANT	59.97	8.87E-12	0.97	0.46
58	4165	FIRST INSEMINATION/PREGNANT	60.68	8.77E-12	0.97	0.46
59	4165	FIRST INSEMINATION/PREGNANT	59.13	8.67E-12	0.97	0.47
60	4165	FIRST INSEMINATION/PREGNANT	60.11	8.66E-12	0.97	0.47
61	4165	FIRST INSEMINATION/PREGNANT	60.32	8.52E-12	0.98	0.48
62	4165	FIRST INSEMINATION/PREGNANT	58.89	8.59E-12	0.98	0.46
63	4165	FIRST INSEMINATION/PREGNANT				
64	4165	FIRST INSEMINATION/PREGNANT	60.00	8.59E-12	0.97	0.49
65	4165	FIRST INSEMINATION/PREGNANT	60.09	8.62E-12	0.98	0.48

n = 2

Day	cow number	state	Δε	τ (s)	α	σ (S/m)
1	4151	NON-PREGNANT	60.15	8.21E-12	0.95	0.44
2	4151	NON-PREGNANT	57.64	8.11E-12	0.98	0.44

3	4151	NON-PREGNANT	63.35	8.12E-12	0.96	0.43
4	4151	NON-PREGNANT	54.81	8.64E-12	0.98	0.38
5	4151	NON-PREGNANT	57.87	8.2E-12	0.98	0.39
6	4151	NON-PREGNANT	61.39	8.54E-12	0.96	0.46
7	4151	NON-PREGNANT				
8	4151	NON-PREGNANT	58.54	8.15E-12	0.97	0.39
9	4151	NON-PREGNANT	58.74	8.56E-12	0.97	0.38
10	4151	NON-PREGNANT	56.48	7.08E-12	0.99	0.29
11	4151	NON-PREGNANT	54.59	8.24E-12	0.97	0.35
12	4151	NON-PREGNANT	59.04	8.26E-12	0.98	0.40
13	4151	NON-PREGNANT	59.39	8.45E-12	0.97	0.41
14	4151	NON-PREGNANT				
15	4151	FIRST INSEMINATION/PREGNANT	56.10	8.31E-12	0.98	0.33
16	4151	FIRST INSEMINATION/PREGNANT	58.37	8.99E-12	0.97	0.38
17	4151	FIRST INSEMINATION/PREGNANT	53.08	7.14E-12	1.00	0.32
18	4151	FIRST INSEMINATION/PREGNANT	58.05	7.53E-12	0.99	0.35
19	4151	FIRST INSEMINATION/PREGNANT	57.93	7.86E-12	0.98	0.34
20	4151	FIRST INSEMINATION/PREGNANT	58.10	8.58E-12	0.98	0.39
21	4151	FIRST INSEMINATION/PREGNANT				
22	4151	FIRST INSEMINATION/PREGNANT				
23	4151	FIRST INSEMINATION/PREGNANT	60.20	8.73E-12	0.97	0.41
24	4151	FIRST INSEMINATION/PREGNANT	57.07	9.2E-12	0.98	0.37
25	4151	FIRST INSEMINATION/PREGNANT	58.97	8.93E-12	0.98	0.41
26	4151	FIRST INSEMINATION/PREGNANT	58.07	8.91E-12	0.98	0.40
27	4151	FIRST INSEMINATION/PREGNANT	59.50	8.79E-12	0.98	0.40
28	4151	FIRST INSEMINATION/PREGNANT				

29	4151	FIRST INSEMINATION/PREGNANT	60.67	7.22E-12	0.99	0.30
30	4151	FIRST INSEMINATION/PREGNANT	60.52	9.23E-12	0.95	0.35
31	4151	FIRST INSEMINATION/PREGNANT	58.44	8.37E-12	0.97	0.38
32	4151	FIRST INSEMINATION/PREGNANT	58.08	7.93E-12	0.98	0.37
33	4151	FIRST INSEMINATION/PREGNANT				
34	4151	FIRST INSEMINATION/PREGNANT	59.97	7.97E-12	0.98	0.39
35	4151	FIRST INSEMINATION/PREGNANT				
36	4151	FIRST INSEMINATION/PREGNANT	60.74	9.08E-12	0.96	0.44
37	4151	FIRST INSEMINATION/PREGNANT	59.79	8.99E-12	0.97	0.42
38	4151	FIRST INSEMINATION/PREGNANT	57.42	9.16E-12	0.97	0.39
39	4151	FIRST INSEMINATION/PREGNANT	59.33	9.3E-12	0.97	0.42
40	4151	FIRST INSEMINATION/PREGNANT	59.23	9.09E-12	0.97	0.43
41	4151	FIRST INSEMINATION/PREGNANT	58.00	9.09E-12	0.97	0.41
42	4151	FIRST INSEMINATION/PREGNANT				
43	4151	FIRST INSEMINATION/PREGNANT	58.63	9.19E-12	0.97	0.40
44	4151	FIRST INSEMINATION/PREGNANT				
45	4151	FIRST INSEMINATION/PREGNANT	59.40	9.07E-12	0.96	0.41
46	4151	FIRST INSEMINATION/PREGNANT	61.11	9.03E-12	0.96	0.44
47	4151	FIRST INSEMINATION/PREGNANT	57.87	9.18E-12	0.97	0.42
48	4151	FIRST INSEMINATION/PREGNANT	59.63	8.21E-12	0.97	0.41
49	4151	FIRST INSEMINATION/PREGNANT				
50	4151	FIRST INSEMINATION/PREGNANT				
51	4151	FIRST INSEMINATION/PREGNANT	60.37	8.45E-12	0.97	0.44
52	4151	FIRST INSEMINATION/PREGNANT	58.87	8.65E-12	0.98	0.41
53	4151	FIRST INSEMINATION/PREGNANT	59.30	8.42E-12	0.97	0.40
54	4151	FIRST INSEMINATION/PREGNANT	58.19	8.88E-12	0.97	0.39

55	4151	FIRST INSEMINATION/PREGNANT	59.22	8.73E-12	0.97	0.42
56	4151	FIRST INSEMINATION/PREGNANT				
57	4151	FIRST INSEMINATION/PREGNANT	60.46	8.72E-12	0.97	0.41
58	4151	FIRST INSEMINATION/PREGNANT	59.35	8.74E-12	0.97	0.42
59	4151	FIRST INSEMINATION/PREGNANT	58.57	8.47E-12	0.97	0.41
60	4151	FIRST INSEMINATION/PREGNANT	57.82	8.71E-12	0.98	0.40
61	4151	FIRST INSEMINATION/PREGNANT	59.05	8.56E-12	0.98	0.42
62	4151	FIRST INSEMINATION/PREGNANT	58.83	8.79E-12	0.97	0.39
63	4151	FIRST INSEMINATION/PREGNANT				
64	4151	FIRST INSEMINATION/PREGNANT	60.57	8.8E-12	0.98	0.42
65	4151	FIRST INSEMINATION/PREGNANT	60.42	8.66E-12	0.97	0.44

n = 3

Day	cow number	state	Δε	τ (s)	α	σ (S/m)
1	3936	NON-PREGNANT	57.46	8.54E-12	0.97	0.58
2	3936	NON-PREGNANT	54.80	7.44E-12	0.97	0.56
3	3936	NON-PREGNANT	61.52	8.59E-12	0.96	0.51
4	3936	NON-PREGNANT	60.89	9.23E-12	0.98	0.47
5	3936	NON-PREGNANT	61.20	8.32E-12	0.98	0.48
6	3936	NON-PREGNANT	59.54	8.60E-12	0.96	0.46
7	3936	NON-PREGNANT				
8	3936	NON-PREGNANT	60.22	8.37E-12	0.97	0.47
9	3936	NON-PREGNANT	60.29	8.01E-12	0.98	0.47
10	3936	NON-PREGNANT	58.30	6.87E-12	1.00	0.39

11	3936	NON-PREGNANT	59.52	8.26E-12	0.97	0.45
12	3936	NON-PREGNANT	59.77	7.88E-12	0.98	0.47
13	3936	FIRST INSEMINATION	59.93	8.21E-12	0.97	0.48
14	3936	FIRST INSEMINATION				
15	3936	FIRST INSEMINATION				
16	3936	FIRST INSEMINATION	61.65	9.49E-12	0.97	0.47
17	3936	FIRST INSEMINATION	56.41	7.11E-12	1.00	0.35
18	3936	FIRST INSEMINATION	59.14	7.98E-12	0.99	0.42
19	3936	FIRST INSEMINATION	59.02	7.51E-12	0.99	0.46
20	3936	FIRST INSEMINATION	62.26	8.46E-12	0.96	0.46
21	3936	FIRST INSEMINATION				
22	3936	FIRST INSEMINATION				
23	3936	FIRST INSEMINATION	60.17	8.65E-12	0.98	0.47
24	3936	FIRST INSEMINATION	57.48	8.87E-12	0.98	0.46
25	3936	FIRST INSEMINATION	60.76	8.95E-12	0.98	0.49
26	3936	FIRST INSEMINATION	59.82	9.18E-12	0.98	0.47
27	3936	FIRST INSEMINATION	55.62	8.77E-12	0.98	0.45
28	3936	FIRST INSEMINATION				
29	3936	FIRST INSEMINATION	60.77	7.56E-12	0.98	0.44
30	3936	FIRST INSEMINATION	60.96	7.91E-12	0.97	0.42
31	3936	FIRST INSEMINATION	58.23	7.99E-12	0.98	0.46
32	3936	FIRST INSEMINATION	57.73	8.10E-12	0.98	0.44
33	3936	FIRST INSEMINATION	59.89	7.77E-12	0.98	0.48
34	3936	FIRST INSEMINATION	58.67	7.64E-12	0.99	0.46
35	3936	FIRST INSEMINATION				
36	3936	FIRST INSEMINATION	62.11	8.83E-12	0.97	0.51

37	3936	FIRST INSEMINATION	59.80	8.88E-12	0.97	0.52
38	3936	SECOND INSEMINATION	59.43	8.83E-12	0.97	0.48
39	3936	SECOND INSEMINATION	60.10	9.07E-12	0.97	0.47
40	3936	SECOND INSEMINATION	60.66	9.26E-12	0.96	0.48
41	3936	THIRD INSEMINATION	58.40	9.02E-12	0.97	0.47
42	3936	THIRD INSEMINATION				
43	3936	THIRD INSEMINATION	61.18	9.23E-12	0.97	0.5
44	3936	THIRD INSEMINATION				
45	3936	THIRD INSEMINATION	62.06	9.21E-12	0.97	0.51
46	3936	THIRD INSEMINATION	56.82	8.97E-12	0.97	0.45
47	3936	THIRD INSEMINATION	60.17	9.28E-12	0.97	0.49
48	3936	THIRD INSEMINATION	59.82	8.50E-12	0.97	0.45
49	3936	THIRD INSEMINATION				
50	3936	THIRD INSEMINATION				
51	3936	THIRD INSEMINATION	61.46	8.49E-12	0.97	0.47
52	3936	THIRD INSEMINATION	60.54	8.65E-12	0.98	0.49
53	3936	THIRD INSEMINATION	60.46	8.43E-12	0.97	0.48
54	3936	THIRD INSEMINATION	60.08	8.68E-12	0.97	0.47
55	3936	THIRD INSEMINATION	59.67	8.64E-12	0.97	0.47
56	3936	THIRD INSEMINATION				
57	3936	THIRD INSEMINATION	58.81	8.70E-12	0.98	0.46
58	3936	THIRD INSEMINATION	59.66	8.65E-12	0.97	0.49
59	3936	THIRD INSEMINATION	61.56	8.63E-12	0.95	0.52
60	3936	THIRD INSEMINATION	60.89	8.38E-12	0.97	0.5
61	3936	THIRD INSEMINATION	60.40	8.37E-12	0.97	0.47
62	3936	THIRD INSEMINATION	63.44	8.59E-12	0.97	0.49

63	3936	THIRD INSEMINATION				
64	3936	THIRD INSEMINATION	59.84	8.78E-12	0.97	0.49
65	3936	THIRD INSEMINATION	59.48	8.77E-12	0.98	0.48

n = 4

Day	cow number	state	Δε	τ (s)	α	σ (S/m)
1	4176	NON-PREGNANT	61.16	8.38E-12	0.94	0.45
2	4176	NON-PREGNANT	58.56	8.11E-12	0.98	0.44
3	4176	NON-PREGNANT	65.12	8.30E-12	0.96	0.43
4	4176	FIRST INSEMINATION	59.84	9.18E-12	0.95	0.41
5	4176	FIRST INSEMINATION	56.46	8.33E-12	0.98	0.38
6	4176	FIRST INSEMINATION	58.41	8.87E-12	0.96	0.40
7	4176	FIRST INSEMINATION				
8	4176	FIRST INSEMINATION	59.02	8.30E-12	0.97	0.40
9	4176	FIRST INSEMINATION	59.73	8.32E-12	0.97	0.34
10	4176	FIRST INSEMINATION	54.90	6.88E-12	0.99	0.31
11	4176	FIRST INSEMINATION	58.91	8.00E-12	0.97	0.39
12	4176	FIRST INSEMINATION	58.31	7.87E-12	0.98	0.42
13	4176	FIRST INSEMINATION	63.65	8.46E-12	0.97	0.44
14	4176	FIRST INSEMINATION				
15	4176	FIRST INSEMINATION	58.64	8.30E-12	0.97	0.39
16	4176	FIRST INSEMINATION	59.50	8.86E-12	0.97	0.42
17	4176	FIRST INSEMINATION	56.09	7.16E-12	1.00	0.30
18	4176	FIRST INSEMINATION	57.36	7.76E-12	0.98	0.36
19	4176	FIRST INSEMINATION	58.71	7.78E-12	0.98	0.39

20	4176	FIRST INSEMINATION	55.01	8.56E-12	0.97	0.38
21	4176	FIRST INSEMINATION				
22	4176	FIRST INSEMINATION				
23	4176	FIRST INSEMINATION	58.44	8.87E-12	0.98	0.40
24	4176	FIRST INSEMINATION	57.85	8.95E-12	0.97	0.40
25	4176	SECOND INSEMINATION				
26	4176	SECOND INSEMINATION	57.58	9.03E-12	0.98	0.40
27	4176	SECOND INSEMINATION	51.67	8.84E-12	0.98	0.34
28	4176	SECOND INSEMINATION				
29	4176	SECOND INSEMINATION	59.16	7.49E-12	0.98	0.36
30	4176	SECOND INSEMINATION	58.79	8.23E-12	0.96	0.35
31	4176	SECOND INSEMINATION	57.16	8.15E-12	0.98	0.38
32	4176	SECOND INSEMINATION	55.60	8.22E-12	0.98	0.36
33	4176	SECOND INSEMINATION	59.06	7.86E-12	0.98	0.40
34	4176	SECOND INSEMINATION	59.10	7.76E-12	0.98	0.40
35	4176	SECOND INSEMINATION				
36	4176	SECOND INSEMINATION	61.09	8.94E-12	0.97	0.35
37	4176	SECOND INSEMINATION	59.68	8.99E-12	0.96	0.41
38	4176	SECOND INSEMINATION	56.68	9.32E-12	0.97	0.40
39	4176	SECOND INSEMINATION	59.35	9.35E-12	0.97	0.42
40	4176	SECOND INSEMINATION	60.06	9.10E-12	0.97	0.44
41	4176	THIRD INSEMINATION	59.47	9.21E-12	0.97	0.43
42	4176	THIRD INSEMINATION				
43	4176	THIRD INSEMINATION	59.15	9.36E-12	0.96	0.44
44	4176	THIRD INSEMINATION				
45	4176	THIRD INSEMINATION	59.60	8.99E-12	0.97	0.43

46	4176	THIRD INSEMINATION	59.53	9.10E-12	0.97	0.44
47	4176	THIRD INSEMINATION	56.64	9.40E-12	0.97	0.38
48	4176	FOURTH INSEMINATION	58.73	8.27E-12	0.97	0.37
49	4176	FOURTH INSEMINATION				
50	4176	FOURTH INSEMINATION				
51	4176	FOURTH INSEMINATION	57.49	8.55E-12	0.97	0.41
52	4176	FOURTH INSEMINATION	56.94	9.16E-12	0.98	0.40
53	4176	FOURTH INSEMINATION	57.94	8.73E-12	0.98	0.40
54	4176	FOURTH INSEMINATION	54.68	8.65E-12	0.98	0.39
55	4176	FOURTH INSEMINATION	59.69	8.87E-12	0.97	0.41
56	4176	FOURTH INSEMINATION				
57	4176	FOURTH INSEMINATION	60.84	8.73E-12	0.97	0.42
58	4176	FOURTH INSEMINATION	59.20	8.82E-12	0.97	0.41
59	4176	FOURTH INSEMINATION	58.25	8.81E-12	0.97	0.40
60	4176	FOURTH INSEMINATION	59.59	8.33E-12	0.97	0.43
61	4176	FOURTH INSEMINATION	56.37	8.57E-12	0.98	0.40
62	4176	FOURTH INSEMINATION				
63	4176	FOURTH INSEMINATION				
64	4176	FOURTH INSEMINATION	58.74	8.92E-12	0.97	0.41
65	4176	FOURTH INSEMINATION	58.51	8.58E-12	0.97	0.42

n = 5

Day	cow number	state	Δε	τ (s)	α	σ (S/m)
1	4049	NON-PREGNANT	61.08	8.08E-12	0.95	0.48

2	4049	NON-PREGNANT	59.95	8.39E-12	0.98	0.49
3	4049	NON-PREGNANT	61.87	8.25E-12	0.96	0.47
4	4049	NON-PREGNANT	60.12	8.75E-12	0.98	0.43
5	4049	NON-PREGNANT	58.72	8.14E-12	0.98	0.44
6	4049	NON-PREGNANT	60.23	8.55E-12	0.97	0.47
7	4049	NON-PREGNANT				
8	4049	NON-PREGNANT	61.94	8.08E-12	0.97	0.45
9	4049	NON-PREGNANT	60.37	8.22E-12	0.98	0.45
10	4049	NON-PREGNANT	57.53	6.83E-12	0.99	0.35
11	4049	NON-PREGNANT	59.39	7.92E-12	0.98	0.43
12	4049	NON-PREGNANT	58.34	7.90E-12	0.98	0.44
13	4049	FIRST INSEMINATION	58.93	8.40E-12	0.97	0.44
14	4049	FIRST INSEMINATION				
15	4049	FIRST INSEMINATION	58.48	8.25E-12	0.98	0.40
16	4049	FIRST INSEMINATION	59.48	8.52E-12	0.97	0.45
17	4049	FIRST INSEMINATION	59.51	7.27E-12	0.99	0.39
18	4049	FIRST INSEMINATION	58.76	7.25E-12	1.00	0.39
19	4049	FIRST INSEMINATION	58.78	7.63E-12	0.98	0.42
20	4049	FIRST INSEMINATION	59.77	8.51E-12	0.98	0.46
21	4049	FIRST INSEMINATION				
22	4049	FIRST INSEMINATION				
23	4049	FIRST INSEMINATION	60.00	8.78E-12	0.97	0.47
24	4049	FIRST INSEMINATION	59.06	8.89E-12	0.98	0.45
25	4049	FIRST INSEMINATION	58.91	8.83E-12	0.98	0.44
26	4049	FIRST INSEMINATION	60.30	9.20E-12	0.98	0.44

27	4049	FIRST INSEMINATION	61.07	8.84E-12	0.98	0.46
28	4049	FIRST INSEMINATION				
29	4049	FIRST INSEMINATION	58.11	7.53E-12	0.98	0.38
30	4049	FIRST INSEMINATION	58.29	7.85E-12	0.97	0.37
31	4049	FIRST INSEMINATION	58.06	8.22E-12	0.98	0.41
32	4049	FIRST INSEMINATION	57.37	8.06E-12	0.98	0.40
33	4049	FIRST INSEMINATION	57.96	7.70E-12	0.99	0.43
34	4049	FIRST INSEMINATION	57.76	7.59E-12	0.98	0.43
35	4049	FIRST INSEMINATION				
36	4049	FIRST INSEMINATION	61.84	9.43E-12	0.97	0.46
37	4049	FIRST INSEMINATION	58.05	9.10E-12	0.97	0.42
38	4049	SECOND INSEMINATION	60.91	9.07E-12	0.97	0.47
39	4049	SECOND INSEMINATION	58.15	9.06E-12	0.97	0.44
40	4049	SECOND INSEMINATION	59.37	8.86E-12	0.97	0.47
41	4049	SECOND INSEMINATION	57.95	9.12E-12	0.97	0.44
42	4049	SECOND INSEMINATION				
43	4049	SECOND INSEMINATION	60.60	9.25E-12	0.97	0.47
44	4049	SECOND INSEMINATION				
45	4049	SECOND INSEMINATION	60.92	8.99E-12	0.97	0.46
46	4049	SECOND INSEMINATION	61.20	8.86E-12	0.69	0.50
47	4049	SECOND INSEMINATION	60.37	8.92E-12	0.97	0.46
48	4049	SECOND INSEMINATION	60.36	8.24E-12	0.97	0.44
49	4049	SECOND INSEMINATION				
50	4049	SECOND INSEMINATION				
51	4049	SECOND INSEMINATION	57.97	8.19E-12	0.98	0.65

52	4049	SECOND INSEMINATION	57.83	8.82E-12	0.98	0.40
53	4049	SECOND INSEMINATION	59.06	8.41E-12	0.98	0.46
54	4049	SECOND INSEMINATION	59.50	8.66E-12	0.98	0.44
55	4049	SECOND INSEMINATION	57.89	8.60E-12	0.97	0.42
56	4049	SECOND INSEMINATION				
57	4049	SECOND INSEMINATION	60.53	8.71E-12	0.97	0.44
58	4049	SECOND INSEMINATION	56.82	8.82E-12	0.97	0.41
59	4049	SECOND INSEMINATION	57.43	8.63E-12	0.97	0.42
60	4049	SECOND INSEMINATION	58.72	8.35E-12	0.98	0.47
61	4049	SECOND INSEMINATION	55.48	8.47E-12	0.97	0.45
62	4049	SECOND INSEMINATION	59.87	8.89E-12	0.98	0.46
63	4049	SECOND INSEMINATION				
64	4049	SECOND INSEMINATION	60.00	8.76E-12	0.97	0.43
65	4049	SECOND INSEMINATION	60.89	8.75E-12	0.97	0.46

n = 6 / Measured during 34 days, extracted from group due to illness and death

Day	cow number	state	Δε	τ (s)	α	σ (S/m)
1	3916	NON-PREGNANT	63.62	8.08E-12	0.95	0.51
2	3916	NON-PREGNANT	54.26	8.39E-12	0.98	0.55
3	3916	NON-PREGNANT	61.44	8.25E-12	0.96	0.60
4	3916	NON-PREGNANT	59.50	8.75E-12	0.98	0.55
5	3916	NON-PREGNANT	58.65	8.14E-12	0.98	0.55

6	3916	NON-PREGNANT	60.16	8.55E-12	0.96	0.57
7	3916	NON-PREGNANT				
8	3916	NON-PREGNANT	58.64	8.08E-12	0.98	0.55
9	3916	NON-PREGNANT	58.90	8.22E-12	0.98	0.55
10	3916	NON-PREGNANT	57.50	6.83E-12	0.99	0.45
11	3916	NON-PREGNANT	56.59	7.92E-12	0.98	0.52
12	3916	NON-PREGNANT		7.90E-12		
13	3916	FIRST INSEMINATION	59.03	8.40E-12	0.97	0.56
14	3916	FIRST INSEMINATION				
15	3916	FIRST INSEMINATION	55.99	8.25E-12	0.98	0.51
16	3916	FIRST INSEMINATION	55.06	8.52E-12	0.97	0.51
17	3916	FIRST INSEMINATION	52.15	7.27E-12	0.99	0.40
18	3916	FIRST INSEMINATION	56.42	7.25E-12	1.00	0.45
19	3916	FIRST INSEMINATION	58.42	7.63E-12	0.99	0.52
20	3916	FIRST INSEMINATION	55.41	8.51E-12	0.98	0.50
21	3916	FIRST INSEMINATION				
22	3916	FIRST INSEMINATION				
23	3916	FIRST INSEMINATION	56.76	8.78E-12	0.99	0.52
24	3916	FIRST INSEMINATION	57.78	8.89E-12	0.99	0.52
25	3916	FIRST INSEMINATION	63.75	8.83E-12	0.98	0.58
26	3916	FIRST INSEMINATION	54.82	9.20E-12	0.99	0.49
27	3916	FIRST INSEMINATION	54.29	8.84E-12	0.99	0.48
28	3916	FIRST INSEMINATION				
29	3916	FIRST INSEMINATION	45.36	7.53E-12	0.96	0.30
30	3916	FIRST INSEMINATION	58.20	7.85E-12	0.96	0.46

31	3916	FIRST INSEMINATION	56.42	8.22E-12	0.98	0.53
32	3916	FIRST INSEMINATION	48.03	8.06E-12	0.99	0.41
33	3916	FIRST INSEMINATION	55.68	7.70E-12	0.99	0.56
34	3916	FIRST INSEMINATION	58.81	7.59E-12	0.99	0.47

n = 7

Day	cow number	state	Δε	τ (s)	α	σ (S/m)
1	4143	NON-PREGNANT	59.91	8.30E-12	0.95	0.46
2	4143	NON-PREGNANT	59.25	8.14E-12	0.98	0.48
3	4143	NON-PREGNANT	63.54	8.25E-12	0.96	0.48
4	4143	NON-PREGNANT	58.17	8.81E-12	0.98	0.43
5	4143	NON-PREGNANT	56.71	8.19E-12	0.98	0.41
6	4143	NON-PREGNANT	62.26	8.64E-12	0.96	0.45
7	4143	NON-PREGNANT				
8	4143	NON-PREGNANT	58.35	8.11E-12	0.97	0.42
9	4143	NON-PREGNANT	57.05	8.31E-12	0.98	0.41
10	4143	NON-PREGNANT	55.77	6.96E-12	0.99	0.31
11	4143	NON-PREGNANT	56.54	7.91E-12	0.98	0.41
12	4143	NON-PREGNANT	57.86	8.05E-12	0.98	0.43
13	4143	FIRST INSEMINATION	58.31	8.41E-12	0.97	0.44
14	4143	FIRST INSEMINATION				
15	4143	FIRST INSEMINATION	56.47	8.10E-12	0.98	0.38
16	4143	FIRST INSEMINATION	58.33	8.73E-12	0.97	0.42

17	4143	FIRST INSEMINATION	55.91	7.38E-12	0.99	0.30
18	4143	FIRST INSEMINATION	57.81	7.51E-12	0.99	0.38
19	4143	FIRST INSEMINATION	57.32	7.89E-12	0.98	0.41
20	4143	FIRST INSEMINATION	59.04	8.60E-12	0.97	0.42
21	4143	FIRST INSEMINATION				
22	4143	FIRST INSEMINATION				
23	4143	FIRST INSEMINATION	59.64	8.76E-12	0.98	0.46
24	4143	FIRST INSEMINATION	58.65	8.84E-12	0.98	0.44
25	4143	FIRST INSEMINATION	59.15	9.13E-12	0.98	0.43
26	4143	FIRST INSEMINATION	56.55	8.90E-12	0.98	0.42
27	4143	FIRST INSEMINATION	56.75	8.88E-12	0.98	0.42
28	4143	FIRST INSEMINATION				
29	4143	FIRST INSEMINATION	57.48	7.65E-12	0.97	0.37
30	4143	FIRST INSEMINATION	57.59	7.65E-12	0.97	0.37
31	4143	FIRST INSEMINATION	59.13	8.19E-12	0.97	0.38
32	4143	FIRST INSEMINATION	55.07	7.92E-12	0.98	0.39
33	4143	FIRST INSEMINATION	58.89	7.83E-12	0.99	0.41
34	4143	FIRST INSEMINATION	58.64	7.59E-12	0.99	0.41
35	4143	FIRST INSEMINATION				
36	4143	FIRST INSEMINATION	59.63	9.07E-12	0.97	0.46
37	4143	FIRST INSEMINATION	58.86	9.06E-12	0.97	0.45
38	4143	SECOND INSEMINATION	56.57	9.29E-12	0.97	0.43
39	4143	SECOND INSEMINATION	59.09	8.95E-12	0.97	0.46
40	4143	SECOND INSEMINATION	59.12	9.20E-12	0.97	0.45
41	4143	SECOND INSEMINATION	58.62	9.05E-12	0.97	0.45

42	4143	SECOND INSEMINATION				
43	4143	SECOND INSEMINATION	59.11	9.38E-12	0.97	0.45
44	4143	SECOND INSEMINATION				
45	4143	SECOND INSEMINATION	59.58	8.94E-12	0.97	0.48
46	4143	SECOND INSEMINATION	59.49	9.07E-12	0.72	0.47
47	4143	SECOND INSEMINATION	60.48	9.09E-12	0.95	0.43
48	4143	SECOND INSEMINATION	58.45	8.46E-12	0.97	0.42
49	4143	SECOND INSEMINATION				
50	4143	SECOND INSEMINATION				
51	4143	SECOND INSEMINATION	57.97	8.19E-12	0.98	0.44
52	4143	SECOND INSEMINATION	51.10	1.37E-14	0.98	0.39
53	4143	SECOND INSEMINATION	58.89	8.59E-12	0.00	0.44
54	4143	SECOND INSEMINATION	58.50	8.86E-12	0.97	0.44
55	4143	SECOND INSEMINATION	58.28	8.83E-12	0.97	0.45
56	4143	SECOND INSEMINATION				
57	4143	SECOND INSEMINATION	59.60	8.84E-12	0.97	0.45
58	4143	SECOND INSEMINATION	58.56	8.84E-12	0.97	0.46
59	4143	SECOND INSEMINATION	57.82	8.45E-12	0.97	0.43
60	4143	SECOND INSEMINATION	58.89	8.71E-12	0.97	0.44
61	4143	SECOND INSEMINATION	58.85	8.63E-12	0.97	0.44
62	4143	SECOND INSEMINATION	55.50	8.81E-12	0.98	0.41
63	4143	SECOND INSEMINATION				
64	4143	SECOND INSEMINATION	56.82	8.64E-12	0.97	0.44
65	4143	SECOND INSEMINATION	56.82	8.64E-12	0.97	0.44

n = 8

Day	cow number	state	Δε	τ (s)	α	σ (S/m)
1	4058	NON-PREGNANT	56.62	8.19E-12	0.98	0.40
2	4058	NON-PREGNANT	57.48	7.87E-12	0.97	0.49
3	4058	NON-PREGNANT	58.73	8.46E-12	0.96	0.47
4	4058	NON-PREGNANT	57.43	9.27E-12	0.98	0.41
5	4058	NON-PREGNANT	57.81	8.31E-12	0.98	0.43
6	4058	NON-PREGNANT	59.29	8.64E-12	0.96	0.45
7	4058	NON-PREGNANT				
8	4058	NON-PREGNANT	57.62	8.30E-12	0.97	0.43
9	4058	NON-PREGNANT	57.95	8.62E-12	0.98	0.43
10	4058	NON-PREGNANT	55.76	7.00E-12	0.99	0.34
11	4058	NON-PREGNANT	58.35	8.07E-12	0.98	0.43
12	4058	NON-PREGNANT	58.72	8.00E-12	0.97	0.45
13	4058	NON-PREGNANT	58.79	8.13E-12	0.97	0.47
14	4058	NON-PREGNANT				
15	4058	NON-PREGNANT	57.51	8.12E-12	0.96	0.39
16	4058	NON-PREGNANT	59.00	8.59E-12	0.97	0.46
17	4058	NON-PREGNANT	50.67	6.74E-12	1.00	0.28
18	4058	NON-PREGNANT	56.68	7.23E-12	0.99	0.39
19	4058	NON-PREGNANT	57.07	7.55E-12	0.98	0.41
20	4058	NON-PREGNANT	58.48	8.23E-12	0.95	0.41
21	4058	NON-PREGNANT				
22	4058	NON-PREGNANT				

23	4058	NON-PREGNANT	57.09	8.66E-12	0.98	0.45
24	4058	NON-PREGNANT	57.55	8.79E-12	0.98	0.45
25	4058	NON-PREGNANT	57.80	8.87E-12	0.98	0.45
26	4058	NON-PREGNANT	57.78	8.91E-12	0.98	0.45
27	4058	NON-PREGNANT	54.88	8.79E-12	0.98	0.43
28	4058	NON-PREGNANT				
29	4058	NON-PREGNANT	55.31	7.74E-12	0.97	0.38
30	4058	FIRST INSEMINATION	57.50	7.94E-12	0.96	0.39
31	4058	FIRST INSEMINATION	56.62	8.19E-12	0.98	0.40
32	4058	FIRST INSEMINATION	57.00	8.09E-12	0.98	0.40
33	4058	FIRST INSEMINATION	57.61	8.01E-12	0.98	0.42
34	4058	FIRST INSEMINATION	57.06	7.86E-12	0.98	0.39
35	4058	FIRST INSEMINATION				
36	4058	FIRST INSEMINATION	59.22	8.98E-12	0.97	0.46
37	4058	FIRST INSEMINATION	59.09	9.17E-12	0.97	0.45
38	4058	FIRST INSEMINATION	57.14	9.09E-12	0.97	0.45
39	4058	FIRST INSEMINATION	56.97	9.22E-12	0.97	0.43
40	4058	FIRST INSEMINATION	58.16	9.49E-12	0.97	0.46
41	4058	FIRST INSEMINATION	57.38	9.29E-12	0.97	0.45
42	4058	FIRST INSEMINATION				
43	4058	FIRST INSEMINATION	57.94	9.17E-12	0.97	0.45
44	4058	FIRST INSEMINATION				
45	4058	FIRST INSEMINATION	59.01	9.10E-12	0.97	0.46
46	4058	FIRST INSEMINATION	59.10	9.00E-12	0.97	0.46
47	4058	FIRST INSEMINATION	55.41	9.13E-12	0.97	0.42

48	4058	FIRST INSEMINATION	59.12	8.46E-12	0.96	0.42
49	4058	FIRST INSEMINATION				
50	4058	FIRST INSEMINATION				
51	4058	FIRST INSEMINATION	56.50	8.31E-12	0.98	0.45
52	4058	SECOND INSEMINATION	58.57	8.79E-12	0.97	0.44
53	4058	SECOND INSEMINATION	57.50	8.56E-12	0.98	0.42
54	4058	SECOND INSEMINATION	55.81	8.78E-12	0.97	0.40
55	4058	SECOND INSEMINATION	55.06	8.89E-12	0.97	0.38
56	4058	SECOND INSEMINATION				
57	4058	SECOND INSEMINATION	58.07	8.71E-12	0.97	0.44
58	4058	SECOND INSEMINATION	59.52	8.87E-12	0.97	0.44
59	4058	SECOND INSEMINATION	55.47	8.53E-12	0.97	0.42
60	4058	SECOND INSEMINATION	56.79	8.53E-12	0.97	0.42
61	4058	SECOND INSEMINATION	57.90	8.38E-12	0.97	0.45
62	4058	SECOND INSEMINATION	56.98	8.79E-12	0.97	0.42
63	4058	SECOND INSEMINATION				
64	4058	SECOND INSEMINATION	56.50	8.75E-12	0.97	0.44
65	4058	SECOND INSEMINATION	57.48	8.75E-12	0.97	0.44

n = 9

**Day**	**cow number**	**state**	**Δε**	**τ (s)**	**α**	**σ (S/m)**
1	4169	NON-PREGNANT	60.13	8.32E-12	0.95	0.51
2	4169	NON-PREGNANT	60.98	7.96E-12	0.98	0.53

3	4169	NON-PREGNANT	61.95	8.20E-12	0.96	0.50
4	4169	NON-PREGNANT	58.55	8.70E-12	0.98	0.47
5	4169	NON-PREGNANT	59.27	8.23E-12	0.98	0.44
6	4169	NON-PREGNANT	62.98	8.59E-12	0.96	0.48
7	4169	NON-PREGNANT				
8	4169	NON-PREGNANT	58.66	8.18E-12	0.97	0.47
9	4169	NON-PREGNANT	60.16	8.17E-12	0.98	0.49
10	4169	NON-PREGNANT	56.78	7.20E-12	0.99	0.37
11	4169	NON-PREGNANT				
12	4169	NON-PREGNANT	59.97	8.02E-12	0.98	0.47
13	4169	NON-PREGNANT	60.29	8.35E-12	0.97	0.49
14	4169	NON-PREGNANT				
15	4169	NON-PREGNANT	58.51	8.09E-12	0.98	0.46
16	4169	NON-PREGNANT	61.37	8.65E-12	0.97	0.51
17	4169	NON-PREGNANT	54.10	7.09E-12	1.00	0.28
18	4169	NON-PREGNANT	59.91	7.31E-12	1.00	0.45
19	4169	NON-PREGNANT	58.84	7.69E-12	0.98	0.47
20	4169	NON-PREGNANT	58.81	8.46E-12	0.97	0.48
21	4169	NON-PREGNANT				
22	4169	NON-PREGNANT				
23	4169	NON-PREGNANT	59.32	8.70E-12	0.98	0.50
24	4169	NON-PREGNANT	58.54	8.95E-12	0.98	0.50
25	4169	NON-PREGNANT	61.33	8.85E-12	0.97	0.50
26	4169	NON-PREGNANT	57.41	8.84E-12	0.99	0.49
27	4169	NON-PREGNANT	59.29	8.78E-12	0.98	0.49

28	4169	NON-PREGNANT				
29	4169	NON-PREGNANT	60.19	7.77E-12	0.97	0.44
30	4169	NON-PREGNANT	62.16	8.59E-12	0.96	0.45
31	4169	NON-PREGNANT	60.54	8.13E-12	0.98	0.45
32	4169	NON-PREGNANT	58.37	8.05E-12	0.98	0.46
33	4169	NON-PREGNANT	60.01	7.76E-12	0.98	0.48
34	4169	NON-PREGNANT	60.05	7.71E-12	0.98	0.47
35	4169	NON-PREGNANT				
36	4169	NON-PREGNANT	60.44	9.21E-12	0.97	0.51
37	4169	NON-PREGNANT	61.81	9.04E-12	0.97	0.51
38	4169	NON-PREGNANT	59.58	9.16E-12	0.97	0.51
39	4169	NON-PREGNANT	60.51	9.69E-12	0.97	0.48
40	4169	NON-PREGNANT	60.14	8.92E-12	0.97	0.52
41	4169	FIRST INSEMINATION	61.80	9.09E-12	0.97	0.51
42	4169	FIRST INSEMINATION				
43	4169	FIRST INSEMINATION	60.92	9.45E-12	0.97	0.49
44	4169	FIRST INSEMINATION				
45	4169	FIRST INSEMINATION	61.38	8.94E-12	0.97	0.51
46	4169	FIRST INSEMINATION	58.18	8.85E-12	0.97	0.50
47	4169	FIRST INSEMINATION	60.99	9.06E-12	0.97	0.49
48	4169	FIRST INSEMINATION	59.57	8.29E-12	0.97	0.47
49	4169	FIRST INSEMINATION				
50	4169	FIRST INSEMINATION				
51	4169	FIRST INSEMINATION	60.09	8.41E-12	0.98	0.52
52	4169	FIRST INSEMINATION	61.50	8.67E-12	0.97	0.49

53	4169	FIRST INSEMINATION	59.24	8.74E-12	0.98	0.49
54	4169	FIRST INSEMINATION	59.05	8.68E-12	0.98	0.48
55	4169	FIRST INSEMINATION	60.53	8.78E-12	0.98	0.50
56	4169	FIRST INSEMINATION				
57	4169	FIRST INSEMINATION	60.40	8.71E-12	0.98	0.50
58	4169	FIRST INSEMINATION	60.16	8.68E-12	0.98	0.50
59	4169	FIRST INSEMINATION	59.49	8.67E-12	0.97	0.49
60	4169	FIRST INSEMINATION	60.97	8.54E-12	0.97	0.51
61	4169	FIRST INSEMINATION	52.14	8.29E-12	0.98	0.41
62	4169	FIRST INSEMINATION	59.69	8.93E-12	0.98	0.50
63	4169	FIRST INSEMINATION				
64	4169	FIRST INSEMINATION	60.93	8.69E-12	0.97	0.49
65	4169	FIRST INSEMINATION	60.17	8.75E-12	0.98	0.49

n = 10

Day	cow number	state	Δε	τ (s)	α	σ (S/m)
1	4082	NON PREGNANT	59.86	8.58E-12	0.97	0.53
2	4082	NON PREGNANT	60.37	8.28E-12	0.98	0.52
3	4082	NON PREGNANT	57.09	8.30E-12	0.96	0.34
4	4082	NON PREGNANT	57.68	8.68E-12	0.98	0.47
5	4082	NON PREGNANT	57.58	8.40E-12	0.98	0.46
6	4082	NON PREGNANT	60.43	8.61E-12	0.96	0.49

7	4082	NON PREGNANT				
8	4082	NON PREGNANT	61.39	8.08E-12	0.97	0.48
9	4082	NON PREGNANT	61.10	8.23E-12	0.97	0.48
10	4082	NON PREGNANT	58.56	7.43E-12	0.98	0.37
11	4082	NON PREGNANT	59.95	7.92E-12	0.98	0.49
12	4082	NON PREGNANT	57.49	7.92E-12	0.98	0.48
13	4082	NON PREGNANT				
14	4082	NON PREGNANT				
15	4082	NON PREGNANT	55.86	8.06E-12	0.98	0.43
16	4082	NON PREGNANT	59.80	8.49E-12	0.97	0.51
17	4082	FIRST INSEMINATION	59.44	7.27E-12	1.00	0.39
18	4082	FIRST INSEMINATION				
19	4082	FIRST INSEMINATION	57.04	7.45E-12	0.98	0.46
20	4082	FIRST INSEMINATION	59.99	8.67E-12	0.98	0.49
21	4082	FIRST INSEMINATION				
22	4082	FIRST INSEMINATION				
23	4082	FIRST INSEMINATION	61.15	8.70E-12	0.98	0.51
24	4082	FIRST INSEMINATION	58.52	8.92E-12	0.98	0.48
25	4082	FIRST INSEMINATION	60.26	8.98E-12	0.98	0.50
26	4082	FIRST INSEMINATION	59.62	8.78E-12	0.98	0.51
27	4082	FIRST INSEMINATION	59.73	9.04E-12	0.98	0.49
28	4082	FIRST INSEMINATION				
29	4082	FIRST INSEMINATION	58.97	7.55E-12	0.98	0.43
30	4082	FIRST INSEMINATION	59.08	8.16E-12	0.97	0.44
31	4082	FIRST INSEMINATION	58.84	8.08E-12	0.98	0.46

32	4082	FIRST INSEMINATION	57.62	7.93E-12	0.98	0.47
33	4082	FIRST INSEMINATION	59.77	7.60E-12	0.98	0.49
34	4082	FIRST INSEMINATION	59.15	7.78E-12	0.98	0.47
35	4082	FIRST INSEMINATION				
36	4082	FIRST INSEMINATION	60.38	9.04E-12	0.97	0.51
37	4082	FIRST INSEMINATION	59.84	9.15E-12	0.97	0.50
38	4082	FIRST INSEMINATION	55.40	9.04E-12	0.97	0.46
39	4082	SECOND INSEMINATION	58.25	9.98E-12	0.97	0.43
40	4082	SECOND INSEMINATION	59.78	9.24E-12	0.97	0.50
41	4082	SECOND INSEMINATION	61.05	9.07E-12	0.96	0.50
42	4082	SECOND INSEMINATION				
43	4082	SECOND INSEMINATION	60.59	9.22E-12	0.97	0.50
44	4082	SECOND INSEMINATION				
45	4082	SECOND INSEMINATION	61.80	8.88E-12	0.96	0.52
46	4082	SECOND INSEMINATION	60.94	8.79E-12	0.97	0.54
47	4082	SECOND INSEMINATION	59.27	9.14E-12	0.96	0.49
48	4082	SECOND INSEMINATION	60.36	8.13E-12	0.97	0.47
49	4082	SECOND INSEMINATION				
50	4082	SECOND INSEMINATION				
51	4082	SECOND INSEMINATION	60.00	8.56E-12	0.98	0.50
52	4082	SECOND INSEMINATION	60.11	9.00E-12	0.97	0.48
53	4082	SECOND INSEMINATION	59.85	8.70E-12	0.98	0.49
54	4082	SECOND INSEMINATION	57.73	8.62E-12	0.98	0.48
55	4082	SECOND INSEMINATION	59.85	8.79E-12	0.97	0.49
56	4082	SECOND INSEMINATION				

57	4082	SECOND INSEMINATION	61.38	8.79E-12	0.97	0.49
58	4082	SECOND INSEMINATION	60.61	8.90E-12	0.97	0.51
59	4082	SECOND INSEMINATION	59.18	8.42E-12	0.98	0.49
60	4082	SECOND INSEMINATION	57.18	8.52E-12	0.97	0.48
61	4082	SECOND INSEMINATION	63.83	8.59E-12	0.97	0.55
62	4082	SECOND INSEMINATION	59.36	8.68E-12	0.97	0.50
63	4082	SECOND INSEMINATION				
64	4082	SECOND INSEMINATION	59.89	8.75E-12	0.97	0.49
65	4082	SECOND INSEMINATION	59.18	8.64E-12	0.97	0.49

SUPPORTING INFORMATION 3: **Physiological and Microwave Dielectric fitting parameters of milk from randomly selected pregnant and non-pregnant cows.**

Table S3A: Physiological parameters (Somatic cell count -SCC, Fat %, protein%, lactose%, and Milk Fat Globule Average diameter) and Microwave Dielectric parameters (Cole-Cole: dielectric strength- Δε, relaxation time τ(s), broadening parameter-α, and conductivity-σ(Siemens/m) of milk from randomly selected pregnant cows. Each measurement corresponds to a different date.

cow number	state	Δε	τ (s)	α	(σ -S/m)	MFG diameter	SCC	Fat	Protein	Lactose
3782	pregnant	59.44	8.6E-12	0.98	0.40	3.55	40	3.36	3.04	4.59
3954	pregnant	59.20	8.49E-12	0.99	0.40	3.66	84	3.42	3.3	5.08
3602	pregnant	60.24	8.37E-12	0.99	0.43	3.35	1419	3.82	3.55	4.57
3933	pregnant	57.09	8.91E-12	0.99	0.44	3.69	429	3.84	3.13	5.1
3665	pregnant	58.28	8.38E-12	0.99	0.44	4.29	165	3.88	3.35	4.65
3950	pregnant	61.04	8.51E-12	0.99	0.49	2.78	392	3.39	3.18	4.81
3970	pregnant	57.12	8.93E-12	0.98	0.37	3.68	596	4.35	3.17	5.02
3485	pregnant	57.51	8.6E-12	0.99	0.49	3.23	50	3.63	3.34	5.04
3950	pregnant	60.17	8.77E-12	0.99	0.49	3.34	420	2.94	3.25	4.59
3866	pregnant	60.46	8.6E-12	0.98	0.44	3.55	44	3.03	3.32	5.33
3633	pregnant	58.44	8.79E-12	1.00	0.44	3.5	229	4.13	3.56	4.56

3802	pregnant	58.90	8.97E-12	0.99	0.50	3.31	70	3.73	3.04	5.4
3835	pregnant	56.09	8.5E-12	0.99	0.43	3.33	164	4.72	3.57	4.72
3867	pregnant	59.65	8.63E-12	0.98	0.44	3.5	23	2.87	3.02	4.89
3599	pregnant	58.23	8.72E-12	0.99	0.46	3.36	32	3.62	2.99	5.27
3287	pregnant	56.97	8.33E-12	0.99	0.46	3.49	1408	3.94	3.6	4.36
3835	pregnant	55.10	8.82E-12	0.99	0.42	3.11	187	4.55	3.73	4.66
3324	pregnant	55.50	8.43E-12	0.99	0.52	3.23	497	4.38	3.41	4.86
3748	pregnant	53.36	8.48E-12	0.98	0.42	3.06	226	3.39	3.07	5.54
3599	pregnant	50.51	8.91E-12	0.98	0.44	3.5	189	3.58	3.31	4.98
3633	pregnant	54.40	8.85E-12	0.99	0.44	3.32	214	4.26	3.28	5.1
3748	pregnant	59.70	8.61E-12	0.98	0.52	3.6	350	3.47	3.04	5.33
3830	pregnant	59.65	8.90E-12	0.99	0.55	3.6	3250	3.56	3.24	4.77
3818	pregnant	58.92	8.89E-12	0.98	0.46	3.97	78	3.63	3.32	4.84
3768	pregnant	59.84	8.83E-12	0.99	0.41	3.49	89	3.77	3.17	5.17
3827	pregnant	59.36	8.15E-12	1.00	0.40	4.25	508	4.46	3.59	4.52
3801	pregnant	*63.04*	*8.36E-12*	*1.00*	*0.52*	*3.27*	*439*	*2.35*	*3.11*	*4.41*
3324	pregnant	61.05	8.18E-12	1.00	0.51	3.33	900	4.69	4.13	4.1

3592	pregnant	58.21	8.16E-12	1.00	0.42	3.68	252	3.04	3.73	4.66
3827	pregnant	*54.93*	*8.55E-12*	*0.98*	*0.49*	*3.21*	*508*	*3.85*	*3.57*	*3.11*
3664	pregnant	59.25	8.57E-12	0.98	0.46	4.14	2596	3.69	3.04	5.24
3856	pregnant	*54.04*	*8.61E-12*	*0.98*	*0.39*	*3.5*	*93*	*3.7*	*3.27*	*5.25*
3768	pregnant	58.27	8.56E-12	0.98	0.44	3.93	89	3.13	3.11	5.17
3485	pregnant	59.09	8.95E-12	0.98	0.49	3.21	35	3.93	3.53	4.83
3610	pregnant	59.15	8.71E-12	0.99	0.43	3.41	66	4.13	2.88	5.12
3768	pregnant	58.93	8.80E-12	0.98	0.45	3.72	89	3.29	3.09	5
3698	pregnant	57.68	8.83E-12	1.00	0.37	2.94	496	3.7	3.64	4.58
3610	pregnant	57.72	8.55E-12	0.99	0.44	3.9	66	3.37	3.27	5.2
3812	pregnant	57.44	8.28E-12	0.99	0.55	4.19	147	3.9	3.59	4.41
3943	pregnant	55.91	8.62E-12	0.99	0.43	3.18	169	4.52	3.28	5.19
3943	pregnant	57.95	8.44E-12	0.99	0.42	3.45	169	4.54	3.53	5.06
3656	pregnant	60.66	8.65E-12	0.99	0.50	3.27	3945	3.39	3.73	3.86
3656	pregnant	55.59	8.48E-12	0.98	0.53	3.2	3945	3.36	3.73	3.86
3936	pregnant	59.48	8.78E-12	1.00	0.42	3.25	428	3.71	3.4	4.67
3698	pregnant	55.41	8.26E-12	1.00	0.34	3.99	1875	4.8	3.77	4.81

3721	pregnant	59.94	8.30E-12	0.99	0.57	4.06	410	3.51	3.6	4.33
3855	pregnant	59.54	8.70E-12	0.99	0.46	3.44	342	3.3	3.62	4.48
3427	pregnant	58.04	8.16E-12	1.00	0.39	4.07	472	4.22	3.59	4.89
3427	pregnant	60.33	8.59E-12	0.98	0.50	3.9	472	3.67	3.09	3.88
3938	pregnant	58.68	8.79E-12	1.00	0.35	4.101	273	3.55	3.53	4.69

Table S3B: Physiological parameters (Somatic cell count -SCC, Fat %, protein%, lactose%, and Milk Fat Globule Average diameter) and Microwave Dielectric parameters (Cole-Cole: dielectric strength- Δε, relaxation time τ(s), broadening parameter-α, and conductivity-σ(Siemens/m) of milk from randomly selected non- pregnant cows. Each measurement corresponds to a different date.

cow number	state	Δε	τ (s)	α	(σ S/m)	MFG diameter	SCC	Fat	Protein	Lactose
3680	Non-pregnant	58.13	8.6E-12	0.99	0.49	3.5	90	3.93	3.11	5.1
3561	Non-pregnant	59.26	8.53E-12	0.99	0.47	2.79	65	3.58	3.42	5.07
3960	Non-pregnant	58.39	8.84E-12	0.99	0.40	4.17	1021	3.17	3.45	4.71
3678	Non-pregnant	60.01	8.79E-12	0.99	0.49	4.64	214	2.87	3.33	4.67
3968	Non-pregnant	56.45	8.55E-12	0.99	0.42	3.309	189	3.84	3.16	5.19
3926	Non-pregnant	60.13	8.82E-12	1.00	0.41	3.11	60	3.73	3.47	4.71

3401	Non-pregnant	59.58	8.55E-12	0.98	0.48	3.55	5506	3.21	3.3	3.62
3837	Non-pregnant	55.58	8.57E-12	0.99	0.41	3.69	2910	4.41	3.27	5.19
3397	Non-pregnant	57.76	8.83E-12	0.99	0.49	3.34	143	4.67	2.98	3.38
3561	Non-pregnant	60.26	9.06E-12	0.99	0.48	3.24	145	3.95	3.24	5.01
3926	Non-pregnant	56.84	8.6E-12	0.99	0.46	2.9	60	3.79	3.04	5.1
3926	Non-pregnant	58.41	8.57E-12	0.99	0.44	3.09	60	3.94	3.11	5.13
3874	Non-pregnant	57.92	8.62E-12	0.99	0.41	4.33	31	3.49	3.33	5.18
3926	Non-pregnant	59.67	8.96E-12	0.98	0.45	3.18	35	3.95	3.02	5.12
3561	Non-pregnant	58.64	8.63E-12	0.98	0.54	3.4	4362	3.76	3.5	4.31
3940	Non-pregnant	56.32	8.84E-12	1.00	0.47	3.46	596	3.27	3.5	4.84
3926	Non-pregnant	57.05	8.82E-12	0.99	0.43	3.294	35	3.3	3.49	4.86
3551	Non-pregnant	60.03	8.69E-12	0.99	0.54	3.61	271	3.44	3.04	4.67
3837	Non-pregnant	55.31	8.59E-12	0.98	0.40	4.26	89	4.18	3.39	5.39
3431	Non-pregnant	58.23	8.56E-12	0.98	0.47	3.47	1849	4.66	3.23	4.02
3585	Non-pregnant	58.56	8.48E-12	0.99	0.49	3.57	246	3.92	3.3	4.74
3403	Non-pregnant	59.96	8.28E-12	0.99	0.50	4.12	126	3.83	3.68	4.49
3431	Non-pregnant	58.48	8.24E-12	1.00	0.41	4.16	1849	5.73	4.24	4.5

3886	Non-pregnant	59.78	8.58E-12	0.98	0.46	4.36	175	3.8	3.36	5.03
3898	Non-pregnant	59.28	8.61E-12	0.99	0.46	3.35	16	3.79	3.23	5.1
3401	Non-pregnant	60.09	8.72E-12	1.00	0.39	3.76	177	3.88	3.56	4.6
3798	Non-pregnant	55.86	8.72E-12	0.99	0.46	3.75	3138	3.93	3.39	4.94
3898	Non-pregnant	59.86	8.84E-12	0.99	0.44	3.92	609	3.37	3.32	4.84
3882	Non-pregnant	59.69	8.64E-12	0.99	0.44	3.56	21	3.7	3	5.07
3371	Non-pregnant	56.80	8.27E-12	0.99	0.42	3.23	427	3.34	3.45	4.59
3997	Non-pregnant	56.92	8.95E-12	0.99	0.39	4.05	195	3.65	2.91	5.25
3971	Non-pregnant	59.81	8.83E-12	1.00	0.42	3.144	85	3.5	3.58	4.47
3882	Non-pregnant	60.08	8.61E-12	0.98	0.45	3.26	165	3.36	3.19	5.08
3808	Non-pregnant	60.37	8.91E-12	0.98	0.51	4.12	193	2.86	2.92	5.03
3901	Non-pregnant	55.08	8.54E-12	0.98	0.45	4.28	183	2.79	3.04	5.32
3971	Non-pregnant	57.04	8.95E-12	0.99	0.41	3.61	80	3.86	3.02	5.27
3726	Non-pregnant	57.97	8.65E-12	0.98	0.40	3.75	63	3.8	3.34	5.27
3678	Non-pregnant	59.65	8.6E-12	0.98	0.47	3.83	37	3.36	3.46	4.69
3667	Non-pregnant	59.30	8.71E-12	0.99	0.46	3.12	74	3.65	3.29	5.09
3696	Non-pregnant	57.83	8.52E-12	0.98	0.40	4.03	28	3.16	3.15	4.99

3726	Non-pregnant	59.52	8.59E-12	0.98	0.50	4.01	63	3.05	3.23	5.15
3667	Non-pregnant	57.69	8.61E-12	0.98	0.45	3.02	74	3.57	3.59	5.04
3882	Non-pregnant	59.71	8.96E-12	0.98	0.47	3.32	296	3.03	3.3	5.19
3971	Non-pregnant	58.65	8.63E-12	0.98	0.47	3.52	94	3.54	3.22	4.37
3860	Non-pregnant	61.46	8.64E-12	0.98	0.47	2.87	74	2.59	3.18	5.62
3791	Non-pregnant	58.10	8.60E-12	0.97	0.45	3.8	42	3.92	3.31	5.23
3605	Non-pregnant	59.83	8.74E-12	0.99	0.47	3.15	27	3.18	3.29	5.16
3785	Non-pregnant	56.86	8.56E-12	0.98	0.43	4.21	38	3.79	3.34	5.13
3794	Non-pregnant	57.76	8.54E-12	0.98	0.43	3.1	40	4.02	3.06	5.07
3770	Non-pregnant	59.24	8.63E-12	0.98	0.42	3.52	40	3.88	3.57	5.08
3817	Non-pregnant	58.32	8.65E-12	0.98	0.42	2.97	40	3.17	3.1	5.23
3803	Non-pregnant	59.07	8.66E-12	0.99	0.53	3.7	145	4.03	3.01	4.84
3776	Non-pregnant	59.21	8.75E-12	0.99	0.49	4.1	74	3.43	3.27	5.17
3873	Non-pregnant	58.74	8.63E-12	0.99	0.50	3.44	345	3.37	2.26	5.7
3665	Non-pregnant	59.30	8.83E-12	1.00	0.39	4.25	165	3.74	3.34	4.75
3776	Non-pregnant	59.82	8.56E-12	0.98	0.45	3.68	74	3.51	3.08	5.01
3397	Non-pregnant	58.13	8.57E-12	0.99	0.49	2.85	87	4.13	3.31	5.08

3671	Non-pregnant	59.03	8.75E-12	0.99	0.46	3.26	44	3.52	3.3	5.25
3968	Non-pregnant	56.49	8.59E-12	0.99	0.49	3.68	131	3.4	3.26	5.26
3774	Non-pregnant	56.37	8.68E-12	0.98	0.47	3.32	103	4.18	3.55	4.92
3840	Non-pregnant	57.84	8.57E-12	0.98	0.45	3.28	132	3.69	2.82	5.57
3624	Non-pregnant	58.26	8.65E-12	0.98	0.45	3.4	28	3.65	3.71	5.12
3870	Non-pregnant	58.54	8.61E-12	0.99	0.42	3.83	225	4.15	3.62	4.66
3870	Non-pregnant	55.18	8.99E-12	0.99	0.43	3.39	207	4.57	3.22	5.22
3701	Non-pregnant	59.21	8.63E-12	0.99	0.39	3.86	179	3.43	3.42	4.61
3701	Non-pregnant	59.42	8.37E-12	0.99	0.48	2.83	179	3.68	3.55	4.49
3701	Non-pregnant	55.66	8.93E-12	0.99	0.46	3.89	221	3.92	3.19	5.1
